# Use of stable isotope-labelled cells to identify active grazers of picocyanobacteria in ocean surface waters

**DOI:** 10.1111/j.1462-2920.2008.01793.x

**Published:** 2009-02

**Authors:** Jorge Frias-Lopez, Anne Thompson, Jacob Waldbauer, Sallie W Chisholm

**Affiliations:** 1 Department of Civil and Environmental Engineering, Massachusetts Institute of Technology15 Vassar Street, Cambridge, MA 02139, USA; 2 MIT/Woods Hole Joint Program in Biological OceanographyCambridge, MA 02139, USA; 3 MIT/Woods Hole Joint Program in Chemical OceanographyCambridge, MA 02139, USA

## Abstract

*Prochlorococcus* and *Synechococcus* are the two most abundant marine cyanobacteria. They represent a significant fraction of the total primary production of the world oceans and comprise a major fraction of the prey biomass available to phagotrophic protists. Despite relatively rapid growth rates, picocyanobacterial cell densities in open-ocean surface waters remain fairly constant, implying steady mortality due to viral infection and consumption by predators. There have been several studies on grazing by specific protists on *Prochlorococcus* and *Synechococcus* in culture, and of cell loss rates due to overall grazing in the field. However, the specific sources of mortality of these primary producers in the wild remain unknown. Here, we use a modification of the RNA stable isotope probing technique (RNA-SIP), which involves adding labelled cells to natural seawater, to identify active predators that are specifically consuming *Prochlorococcus* and *Synechococcus* in the surface waters of the Pacific Ocean. Four major groups were identified as having their 18S rRNA highly labelled: Prymnesiophyceae (Haptophyta), Dictyochophyceae (Stramenopiles), *Bolidomonas* (Stramenopiles) and Dinoflagellata (Alveolata). For the first three of these, the closest relative of the sequences identified was a photosynthetic organism, indicating the presence of mixotrophs among picocyanobacterial predators. We conclude that the use of RNA-SIP is a useful method to identity specific predators for picocyanobacteria *in situ*, and that the method could possibly be used to identify other bacterial predators important in the microbial food-web.

## Introduction

The mechanisms that regulate microbial communities are a central issue in ocean ecology. Phagotrophic protists and viruses are the main sources of mortality for these microbes in oligotrophic environments (Fuhrman and Campbell, 1998; Partensky *et al*., 1999a) and play an important role in shaping microbial communities in the ocean (so-called ‘top-down’ regulation) (Sherr and Sherr, 2002; Pernthaler, 2005). One of the outstanding questions is precisely how the food-web is structured: which protists eat which microbes?

Grazing activity by eukaryotes is a major factor of bacterial mortality in the ocean and a major force for shaping microbial communities in those environments (Jurgens and Matz, 2002). Heterotrophic nanoflagellates and ciliates are considered to be the primary grazers on planktonic marine bacteria (Sherr *et al*., 1989; Simek and Chrzanowski, 1992; Cho *et al*., 2000; Sherr and Sherr, 2002). In general, grazing by bacterivorous protists upon suspended bacteria is size selective (Chrzanowski and Símek, 1990; Gonzalez *et al*., 1990; Simek and Chrzanowski, 1992; Jürgens and Güde, 1994; Anderson and Rivkin, 2001) with most protists grazing preferentially on medium-sized bacterial cells.

Because *Prochlorococcus* and *Synechococcus* numerically dominate the oxygenic phototrophs in ocean waters (Chisholm *et al*., 1988; Partensky *et al*., 1999a,b), understanding their sources of mortality is central to understanding the structure of the microbial food-web, and the regulation of marine productivity and nutrient cycling in the ocean. Laboratory studies using cultured heterotrophic flagellates and ciliates have shown that they can survive when fed *Prochlorococcus* and *Synechococcus* (Christaki *et al*., 1999; Guillou *et al*., 2001) and that some feed preferentially on one or the other (Christaki *et al*., 1999; Guillou *et al*., 2001). Studies using natural nanoflagellate populations show that the nanoflagellate community composition shapes the picoautotrophic community structure and, vice versa, the picoautotrophic community structure favours or inhibits the growth of some nanoflagellates groups (Christaki *et al*., 2005). However, these studies do not address the question of the identity of the grazers feeding on bacteria.

While rates of grazing-induced mortality of picocyanobacteria have been measured *in situ* (Sherr *et al*., 1987; Hall *et al*., 1993; Ishii *et al*., 2002; Massana *et al*., 2002; Worden and Binder, 2003; An-Yi *et al*., 2007), the specific identity of the grazers feeding on these cells has not been studied. In the present work, we have used a modification of a RNA stable isotope probing technique (RNA-SIP) (Radajewski *et al*., 2000; Manefield *et al*., 2002; Lueders *et al*., 2004) to identify eukaryotic cells that consume *Prochlorococcus* and *Synechococcus* in surface waters at the Hawaii Ocean Time Series (HOT) station ALOHA. A similar approach had been previously used to identify micropredators of *Escherichia coli* in a sample of agricultural soil (Lueders *et al*., 2006). The use of this method avoids problems associated with using non-active bacteria (González *et al*., 1990; Landry *et al*., 1991; del Giorgio *et al*., 1996; Ishii *et al*., 2002; Koton-Czaarnecka and Chrost, 2003), and enables molecular taxonomic resolution.

## Results and discussion

### Characterization of the indigenous eukaryotic protist community

**Fig. 1:**
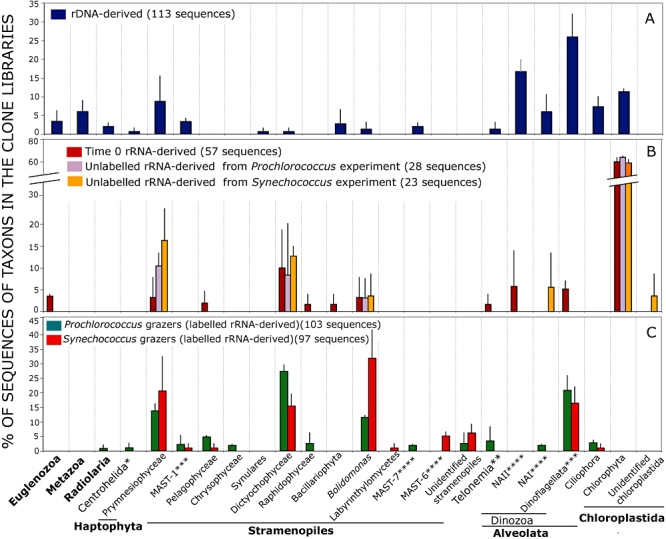
Phylogenetic assignments and relative frequencies of the rDNA sequences from indigenous eukaryotic community, and the labelled and unlabelled rRNA fractions in the experimental treatments. A. rDNA extracted from the total community. B. Unlabelled fractions from the density gradient separations and time 0 samples. C. Samples with label originating from *Prochlorococcus* or *Synechococcus* added to the experimental bottles. Error bars represent the standard deviation of the values obtained for the biological duplicates of the libraries. Phylogenetic assignment follows Adl and colleagues (2005) with classification at the first- (in bold) and second-rank taxonomic level except when indicated as follows: *Super-groups, **Phylum, ***third-rank taxonomic level and ****novel Alveolate groups I and II (NAI and NAII), or the novel MAST following Not and colleagues (2007).

**Fig. 2:**
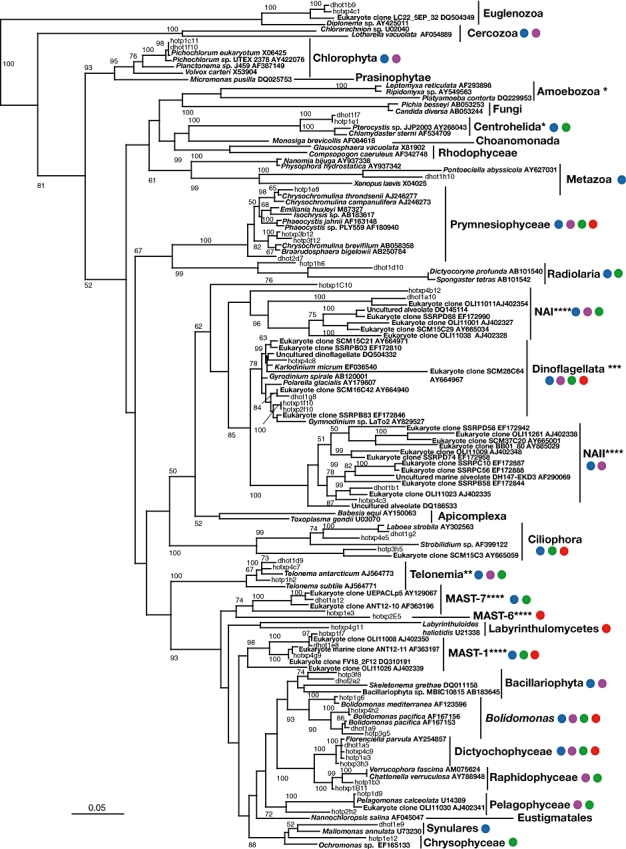
Unrooted phylogenetic tree inferred by maximum likelihood (ML) analysis of the reference sequences used in the phylogenetic analysis of the clone libraries presented in this work (see *Supporting information*). Selected representative clones and colour circles indicate the phylogenetic adscription of the sequences obtained in the different clone libraries. Blue clones and circles: sequences originating from the DNA-derived libraries. Purple clones and circles: sequences originating in the unlabelled fractions from the density gradient separations and time 0 samples. Green clones and circles: sequences originating from the labelled fraction of the *Prochlorococcus* inoculation experiment. Red clones and circles: sequences originating from the labelled fraction from the *Synechococcus* inoculation experiment. Partial sequences ranging from a minimum of 604 bp up to 827 bp were used in the alignment. Bootstrap values over 50% are indicated on the internal branches obtained from Bootstrap values < 50%, which have been omitted. The proportion of invariant sites (*I*) was 0.214. The scale bar indicates 5% divergence. Classification is based on Adl and colleagues (2005) and Not and colleagues (2007). All groups correspond to first and second rank according to Adl and colleagues (2005) except *Super-group and **Phylum (Shalchian-Tabrizi *et al*., 2006), ***third-rank taxonomic level and ****novel Alveolate groups I and II (NAI and NAII), or the novel MAST following Not and colleagues (2007).

We first characterized the diversity of protists in our sample, collected from the study site, Station ALOHA (Hawaii Ocean Time Series) through the analysis of the indigenous 18S rDNA sequences (Figs 1A and 2 and Fig. S1). The community was similar to those reported for other oligotrophic surface ocean waters (Countway *et al*., 2005; 2007; Not *et al*., 2007), in terms of first- and second-rank marine protistan and Super-group taxa defined by Adl and colleagues (2005). Alveolates, and specifically Dinozoa, including novel Alveolate groups I and II (NAI and NAII), are among the most abundant sequences found. Stramenopiles, including novel Marine Stramenopiles (MAST), are also well represented (Figs 1A and 2and Fig. S1).

### Incubation experiments with labelled cultures

**Fig. 3:**
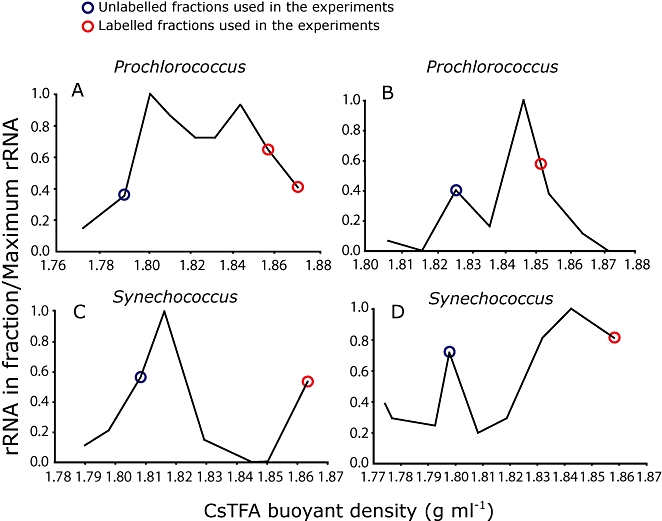
Relative amount of rRNA in different fractions separated by density gradient centrifugation of 18S rRNA analysed in this study. Two peaks of RNA were detected in each sample, the lighter containing sequences that did not incorporate the isotopic label (^15^N and ^13^C) during the 24 h incubation, and the heavier to the RNA greatly enriched in the heavy isotope, i.e. from cells that incorporated label from the *Prochlorococcus* and *Synechococcus* that were added to the samples. The particular samples indicated in a blue circle were analysed as the ‘unlabelled fraction’ from the experimental bottles, and those in a red circle the ‘heavily labelled’ fraction. These particular samples were chosen to maximize the sample size while at the same time avoiding cross-contamination of light and heavy RNA in the subsequent analyses. (A) and (B) represent sequences from biological replicates of samples amended with labelled *Prochlorococcus* (in A two heavy fractions were used to increase the amount of total RNA used for constructing the clone libraries) and (C) and (D) those amended with *Synechococcus*. Total RNA was detected fluorometrically using Ribogreen (see *Experimental procedures*).

To determine which protists from this community most actively grazed on *Prochlorococcus* and *Synechococcus*, ^13^C- and ^15^N-labelled cultures of these cyanobacteria were added to seawater samples and incubated for 1 day, allowing the indigenous community to consume the labelled cells (see *Experimental procedures* for details). After 24 h, the microbial community was collected by filtration, RNA was extracted, and ‘heavy’ (labelled) and ‘light’ (unlabelled) RNA was separated by density gradient ultracentrifugation. Density-resolved 18S rRNA sequences were amplified, sequenced and analysed. Sequences from the labelled subfraction (which are enriched in a subset of sequences as they are physically separated from the bulk community before sequencing) are interpreted as being derived from eukaryotic cells that consumed high numbers of labelled *Prochlorococcus* or *Synechococcus* cells during the incubation. Sequences in the unlabelled RNA fraction represent protists that did not graze on the labelled cells during the incubation. Because different levels of RNA labelling are likely to occur depending on what fraction of the diet of a particular grazer consists of *Prochlorococcus* and *Synechococcus*, we analysed only the most highly labelled fractions (Fig. 3).

We recognize that there are, theoretically, a number of possible indirect routes for the heavy isotopes to end up in the 18S rRNA. We analysed these possibilities in detail in a separate section below, and conclude that direct grazing on *Prochlorococcus* and *Synechococcus* is the most consistent explanation for the incorporation of label into 18S rRNA in our experiments.

### Community structure analysis using terminal restriction fragment length polymorphism (T-RFLP)

**Fig. 4:**
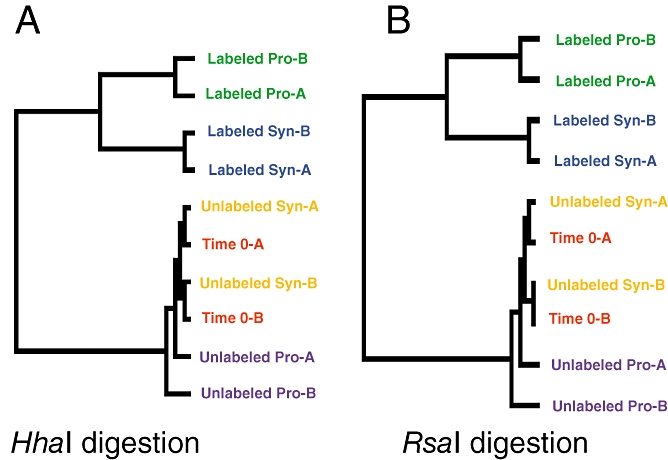
Self-organizing tree (SOTA) of terminal restriction fragment length polymorphism (T-RFLP) profiles emerging from the 18S rRNA sequences from the different experimental treatments. Samples digested (A) with HhaI and (B) with RsaI. Time 0: T-RFLP profile of rRNA from the entire eukaryotic community at the beginning of the experiment. Unlabelled Pro and Unlabelled Syn: T-RFLP profiles of the unlabelled eukaryotic rRNA, collected from the density gradient, from the bottles that were incubated with labelled *Prochlorococcus* and *Synechococcus* respectively. Labelled Pro and Labelled Syn*:* T-RFLP profiles of the heavily labelled eukaryotic rRNA that was collected from the same gradient. A and B next to the data points represent the two biological replicates.

Before analysing the sequences of rRNA from the labelled and unlabelled fractions in detail, we assessed the quality of the biological replicates and general differences and similarities among the treatments, using terminal restriction length polymorphism (T-RFLP) and cluster analysis (GEPAS, http://www.gepas.org) (Dollhopf *et al*., 2001). The eukaryotic cells at the onset of the experiment (time 0), as well as those that remained unlabelled after a 24 h incubation (i.e. those that did not prey on either *Prochlorococcus* or *Synechococcus*), cluster together in both replicates (Fig. 4). The similarity of these two groups indicates that there were no significant changes in the food-web structure in the incubation bottles during the 24 h incubation. More importantly, the 18S rRNA sequences containing the *Prochlorococcus*-derived label and *Synechococcus*-derived label clustered separately from the time 0 and unlabelled rRNA samples, indicating that we are identifying a specific subset of the community that is preying upon these cyanobacteria. Furthermore, the predator sequences originating from addition of *Prochlorococcus* and *Synechococcus* did not cluster together, suggesting distinct predators for these two types of cyanobacteria, consistent with observations from laboratory studies (Guillou *et al*., 2001; Pernthaler, 2005).

### Analysis of the unlabelled and labelled 18S rRNA sequences

We next analysed the identity of the unlabelled and labelled eukaryotes by cloning and sequencing the 18S rRNA fragments from the heavily labelled and unlabelled fractions isolated from the density gradient separation (Fig. 3). Heavily labelled fractions represent eukaryotes that have eaten either *Prochlorococcus* or *Synechococcus*. The unlabelled fractions represent eukaryotes in the community with relatively high levels of rRNA that did not assimilate label from the cyanobacteria. As has been reported previously (Stoeck *et al*., 2007) the sequences in the unlabelled rRNA-derived library are substantially different from those in the rDNA library (Figs 1A and B and 2; Figs S1 and S2), showing that there are some members of the community that are much more ‘active’ (as measured by rRNA levels) than others.

Most sequences obtained in the rRNA-derived library from the time 0 samples and unlabelled fractions represented members of the Chlorophyta, principally close relatives of the genus *Picochlorum* (Figs 1B and 2 and Fig. S2). Other taxonomic groups identified in these libraries included the Dictyochophyceae (Stramenopiles) and Prymnesiophyceae (Haptophyta), and in smaller numbers relatives of members of Raphidophyceae, *Bolidomonas*, Bacillariophyta, Pelagophyceae (all Stramenopiles), Euglenozoa and Dinozoa (Alveolota) (Figs 1B and 2 and Fig. S2).

The sequences that appeared in the labelled fractions (Figs 1C and 2, Figs S3 and S4) – i.e. from cells grazing on *Prochlorococcus* and *Synechococcus*– belonged primarily to four groups: the Prymnesiophyceae, Dictyochophyceae, *Bolidomonas* and Dinoflagellata. Dictyochophyceae dominated the 18S rRNA sequences that had incorporated label from *Prochlorococcus*, while *Bolidomonas* dominated those that had incorporated the label from *Synechococcus* (Fig. 1C), but it appears that the four dominant grazers consume both types of cells. Novel MAST also appeared in both labelled rRNA-derived libraries and they have been identified as non-pigmented heterotrophic flagellates with bacterivory activity (Massana *et al*., 2002). Some taxonomic groups appear to be specific to either *Prochlorococcus* or *Synechococcus* (Figs 1C and 2, Figs S3 and S4), but this could be simply due to the small library sample size. Certain groups that were present in the labelled rRNA-derived clone libraries but were absent in the unlabelled rRNA-derived clone libraries could have been simply masked by the high dominance of Chlorophyta in the rRNA-derived clone libraries in relation to the rest of identified phylogenetic groups.

Ciliates, which are considered important grazers in some aquatic environments (Sherr and Sherr, 2002; Pernthaler, 2005), represent a small fraction of the labelled sequences, which is consistent with recent work showing that subtropical marine ciliates exhibit almost no grazing activity on bacterium-sized particles (An-Yi *et al*., 2007) and with the experimental results by Christaki and colleagues (1999) showing that *Prochlorococcus* and *Synechococcus* proved to be poor food sources for ciliate growth.

The most striking observation in these results is that three of the four most abundant sequences in the labelled 18S rRNA fraction belong to the taxa Prymnesiophyceae, Dictyochophyceae and *Bolidomonas*, whose characterized members are photosynthetic. Two groups present in the labelled 18S rRNA fraction, the Pelagophyceae and *Bolidomonas*, have not previously been found to consume bacteria. Some Pelagophyceae feed heterotrophically on dissolved organic matter (Lomas *et al*., 2001) but this group has previously been described as non-phagotrophic (Cavalier-Smith and Chao, 2006). Characterized members of the *Bolidomonas*, the most frequently detected labelled group in the *Synechococcus*-fed samples (Figs 1C and 2 and Fig. S4), are all photosynthetic. While some members of the Dictyochophyceae, which dominate the clone libraries from the labelled *Prochlorococcus*-fed samples (Figs 1C and 2 and Fig. S3), are heterotrophs, the closest relative to the sequences we have identified is *Florenciella parvula*, which is photosynthetic. Also among the identified predators of *Prochlorococcus* and *Synechococcus* are representatives of groups known to be capable of mixotrophy, including the Chrysophyceae (Nygaard and Tobiesen, 1993), Prymnesiophyceae (Nygaard and Tobiesen, 1993; Hansen and Hjorth, 2002) and Dinoflagellata (Hansen and Nielsen, 1997). Almost all of the sequences from the labelled clone libraries belong to plastid-containing lineages; only sequences identified as relatives of *Telonema* (phylum Telonemia) (Shalchian-Tabrizi *et al*., 2006) and Centrohelida come from groups not known to contain autotrophic members.

Previous work had already presented evidence that mixotrophic nanoflagellates are important predators in surface waters and may make up more than 50% of the bacterivory in them, and that they are more abundant near ocean surface waters than in the deeper euphotic zone (Arenovski *et al*., 1995; Caron, 2000). Moreover, previous studies have demonstrated that pigmented and non-pigmented nanoflagellates had similar grazing rates on heterotrophic bacteria (Hall *et al*., 1993).

### Detection of label in plastid 16S rRNA

To further test the mixotrophy hypothesis we examined whether the labelled fraction contained plastid DNA using primers designed specifically for the 16S rRNA sequence in chloroplast DNA (Table S1). We designed these primers specifically to amplify plastid 16S rRNA genes, but not *Prochlorococcus* and *Synechococcus* 16S rRNA, since the latter would have dominated our signal. This meant we did not recover as many plastid sequences as we might have if we had used published plastid 16S rRNA primers (Fuller *et al*., 2006), but this was an unavoidable limitation, given the experimental design.

Two of the primer sets for plastid 16S rRNA (primers sets 6 and 15, Table S1) yielded PCR products of the expected size, and in the case of primer set 15 the product was long enough (approximately 650 bp) to be sequenced and analysed. Although it is difficult to determine the exact affiliation of these chloroplast sequences given the short length of the amplified PCR product, and the limited coverage of chloroplast sequences from different plastid-containing phylogenetic groups in the database, the phylogenetic analysis showed that the amplified sequences from the labelled fraction were indeed from chloroplasts (Fig. S5). Furthermore, the phylogenetic analysis showed that the closest relatives of the chloroplasts identified in our labelled fraction were related to *Bolidomonas mediterranea* and diatom chloroplasts (Fig. S5). As there are a limited number of chloroplast sequences representing other groups of Stramenopiles in the databases, and given the short size of the analysed product, the exact phylogenetic affiliation of these sequences is not entirely clear. The key finding, however, is that all of the sequences obtained cluster with chloroplasts indicating that the heavy label ended up in eukaryotic cells capable of photosynthesis.

### Analysis of alternative routes for label incorporation

In this and other types of labelling experiments with natural populations, the possibility that the isotopic label might have been acquired by protists via a route other than phagotrophic predation must be considered. For example, it is conceivable that the label might have passed through a dissolved phase, either organic or inorganic, and was acquired through non-phagotrophic nutrient uptake. Alternatively, the label could have been initially acquired by bacterial heterotrophs that were subsequently grazed by phagotrophs. Below we consider each of these possibilities in turn, and present evidence that they do not appear to be playing a role in these experiments.

The labelled cyanobacterial biomass could have been transformed to dissolved inorganic carbon (DIC) through respiration, either by the picocyanobacteria themselves or by other heterotrophs. Had a substantial amount of the added biomass been respired, that labelled carbon would have become broadly available for fixation by all of the autotrophs in the sample, which would then appear in the labelled fraction. In fact, the most abundant sequences in the unlabelled rRNA-derived clone libraries – the photoautotrophic Chlorophyta – were not represented in the labelled fraction (Figs 1B and C and 2, Figs S2–S4). This demonstrates that no significant quantity of labelled DIC was available for photosynthetic fixation, and passage of the label through the dissolved carbonate pool can be excluded.

Another possibility would be that the initially supplied, isotopically labelled biomass might have entered the dissolved organic carbon (DOC) pool by exudation, lysis or ‘sloppy feeding’ by zooplankton. The latter two mechanisms would result in substantial declines in the picocyanobacterial population during the experiment; however, the concentration of picocyanobacteria did not change dramatically over the 24 h of incubation. In all cases the initial and final concentration, after 24 h of incubation, of both *Prochlorococcus* and *Synechococcus* was of 10^5^ cells ml^−1^, suggesting that mechanisms involving cell death (including lysis and sloppy feeding) did not release large amounts of biomass into the dissolved phase. To consider exudation, we can use the *Prochlorococcus* addition experiment as an example. *Prochlorococcus* MED4 cells were added to the seawater sample at a concentration of 1.7 × 10^5^ per ml and typically contain about 60 fg of carbon per cell (Bertilsson *et al*., 2003). If we imagine that the added *Prochlorococcus* could somehow exude all of their initial labelled carbon as DOC – while suffering no great decline in cell numbers – this is equivalent to the addition of 0.9 μM of ^13^C-DOC, clearly an upper limit for the potential contribution of the isotopically labelled *Prochlorococcus* to the DOC pool. Typical surface total DOC concentrations at station ALOHA, where the samples for this study were taken, are around 75 μM, of which 40 μM is likely refractory organic matter that turns over very slowly (Carlson, 2002). Hence there is roughly 35 μM of labile DOC available for rapid heterotrophic consumption. Addition of *Prochlorococcus*-derived ^13^C-DOC to this could result in a 36 μM pool of labile DOC with maximum ^13^C content of 3.5 atom%, which is in turn the upper limit for labelling by DOC consumption. Similar considerations limit the ^13^C content of DOC in the *Synechococcus* addition experiments to 10.6 atom%.

Next, we consider the extent of labelling of the heavy RNA fractions in our incubation experiments. The difference in buoyant density between heavy and light RNA fractions in these experiments ranged from 0.034 to 0.078 g ml^−1^ (Fig. 3), equal to or exceeding the buoyancy differences (0.035–0.04 g ml^−1^) observed by Lueders and colleagues (2004) for 100% ^13^C-labelled SSU rRNA. This large difference in buoyant density suggests that the heavy fractions analysed in this experiment were highly labelled, likely in excess of 90 atom% ^13^C. This is far greater than the 3–11% possible from DOC consumption, even under the assumption of maximally rapid exudation by the added cyanobacteria. The buoyancy differences observed here in excess of the ∼0.4 g ml^−1^ reported by Lueders and colleagues (2004) may reflect ^15^N incorporation and/or differences in centrifugation conditions. In any event, the heavy RNA in these experiments is much too highly labelled to derive from heterotrophic consumption of DOC.

A third, even more mechanistically complicated possibility is the direct and specific consumption of picocyanobacteria by heterotrophic bacteria or the consumption of labelled DOC exuded by, or otherwise released from, the picocyanobacteria by those heterotrophs. Protistan predators can then graze on these labelled heterotrophs. If this occurred, the 18S sequences observed in the heavy fraction would reflect grazing activity, though not specifically on *Prochlorococcus* or *Synechococcus*. Under this scenario, a subset of heterotrophic bacteria would become highly labelled, and their RNA should be found in the heavy fraction. To address this possibility, we constructed 16S rRNA clone libraries as described in *Experimental procedures*. If there had been transfer of labelled organic matter through heterotrophic bacteria at the level needed to fractionate differentially in a CsTFA gradient we would expect to find 16S rRNA sequences from heterotrophic bacteria. Forty-three clones from the labelled fractions were sequenced. Seventeen clones came from the fraction obtained from the bottles inoculated with labelled *Prochlorococcus* MED4 and in all cases the best blastn match for those sequences corresponded to *Prochlorococcus marinus*. Similarly, 26 clones coming from the fraction obtained from the bottles inoculated with labelled *Synechococcus* WH8102 and in all cases the best blastn match corresponded to *Synechococcus*. Additionally, 11 clones coming from the unlabelled fraction from the *Prochlorococcus* experiment were sequenced and 18% of those corresponded to *P. marinus*, while the rest were sequences from heterotrophic bacteria. These results demonstrate that 16S rRNA compositions of the labelled and unlabelled fractions were indeed distinct, and that heterotrophic bacteria did not appear to become highly labelled over the course of the incubation. We thus conclude that the labelled eukaryotes did not obtain their label indirectly via predation of heterotrophic bacteria.

### Conclusions and implications

The reproducibility and internal consistency of the results obtained in the study indicate that the use of RNA-SIP for studying the marine microbial food-webs *in situ* has tremendous potential. There are a multitude of variations on this experimental design that could yield many insights into the specific pathways of the flow of carbon and energy in the marine food-web. These particular results also reveal that a significant fraction of the eukaryotes that we identified as grazing specifically on *Prochlorococcus* and *Synechococcus* were likely mixotrophs – i.e. cells that utilize both phototrophy and phagotrophic heterotrophy as a way of obtaining nutrients and energy (Raven, 1997; Jones, 2000). While a few studies have provided evidence of the importance of mixotrophy in marine aquatic environments (Arenovski *et al*., 1995; An-Yi *et al*., 2007; Unrein *et al*., 2007), this is the first study to identify marine mixotrophs through their grazing activity on specific prey.

The adoption of mixotrophy as a survival strategy under oligotrophic oceanic conditions might confer a fitness advantage for a number of reasons (Raven, 1997). First, phagotrophy may be a way for relatively large eukaryotic cells to acquire inorganic nutrients such as N, P and Fe in oligotrophic waters. Arenovski and colleagues (1995) presented experimental evidence of a decrease in the abundance of mixotrophic phototrophs under nutrient enrichment conditions, suggesting that phagotrophy is used under low dissolved nutrient concentrations, conditions that are normal in surface oligotrophic water. With their larger surface to volume ratio, picocyanobacteria like *Prochlorococcus* and *Synechococcus* likely have an advantage over larger eukaryotic cells in acquiring dissolved nutrients. Consuming cyanobacteria may also be a way for the larger cells to increase their relative fitness by reducing the abundance of their competitors for nutrients. Mixotrophy has been linked to survival of nanoflagellates under nutrient limitation (Unrein *et al*., 2007) and it has been shown that algal flagellates increase bacterivory under phosphate limitation (Nygaard and Tobiesen, 1993). Moreover, the metabolic costs of adding phagotrophic machinery to an otherwise photosynthetic metabolism may be rather low in comparison with the potential benefits (Raven, 1997).

Predation by mixotrophs also has implications for our understanding of the population dynamics of marine picocyanobacteria. While picocyanobacteria are generally the numerically dominant phytoplankton in stratified oligotrophic open-ocean waters, they usually do not bloom (i.e. increase markedly in cell concentrations) in response to episodic nutrient supplies (Mann and Chisholm, 2000). This behaviour has been explained by concomitant increases in grazing rates, implying that these grazers are able to respond very quickly to shifts in prey growth and quality. Our identification of mixotrophic predators may shed further light on this dynamic: eukaryotic mixotrophs directly exploit the same episodic supplies of dissolved nutrients as their picocyanobacterial prey, and thus could grow faster, through stimulated autotrophy, as nutrients become more abundant. As their populations grow and consume the available nutrients, they may shift towards phagotrophy, increasing the mortality rate of cyanobacteria, preventing bloom formation even in the face of rapid growth rates. This hypothesis is directly testable using the approach we have described.

As evidence increasingly points towards the mixotrophic capabilities of both nominally photo- and heterotrophic organisms it is becoming clear that a sharp distinction between photosynthetic and predatory lifestyles is a false dichotomy. It is likely that marine protists utilize a spectrum of trophic strategies, ranging between obligate photoautotrophic and strictly phagotrophic end members and occupying nearly all gradations in between (Sanchez-Puerta *et al*., 2007). Further investigations regarding other ocean sites and different depths are needed to confirm the potential importance of mixotrophy as a common metabolic strategy for grazes feeding on picocyanobacteria.

## Experimental procedures

### Sampling and incubation conditions

*Prochlorococcus* MED4 and *Synechococcus* WH8102 were grown for 4 days at 19°C under continuous cool white light (16.6 μmol Q m^−2^ s^−1^) in artificial seawater medium (Rippka *et al*., 2000) amended with 6 mM ^13^C-sodium bicarbonate and 800 μM ^15^N-ammonium chloride. Cells were harvested by centrifugation at 8000 *g* for 15 min and washed twice in unlabelled artificial seawater medium and re-suspended in the same medium. Cells were counted by flow cytometry to have an estimate of the volume of inoculum to be used in the experiment, in order to have a final concentration of picocyanobacteria similar to the concentration found in natural samples (approximately 10^5^ cells ml^−1^). Final isotopic enrichment of the cultures was measured by mass spectrometry at UC Davis Stable Isotope Facility using on-line combustion (Europa Integra): atom% ^13^C for *Prochlorococcus* MED4 was 98.86% and for *Synechococcus* WH8102 84.20% and atom% ^15^N for *Prochlorococcus* MED4 was 61.13% and for *Synechococcus* WH8102 39.83%. These cultures were then transported overnight in the dark to the field site for use in the grazing experiments.

Samples of ocean surface water (3–5 m depth) were collected in 500 ml acid cleaned bottles during the month of March 2006 as a part of HOT cruise 179, and inoculated with either labelled *Prochlorococcus* MED4 or *Synechococcus* WH8102 at a final concentration of 10^5^ cells ml^−1^. All shipboard incubations were performed in duplicate and analysed independently. The incubations were set in an on-deck incubator, which was constantly re-circulated with surface seawater to maintain temperature. Two samples of 200 ml were collected at the beginning of the experiment as a control to identify the initial eukaryotic community. Samples of 250 ml were collected from the bottles with added labelled *Prochlorococcus* and *Synechococcus* after 24 h of incubation. The 24 h period allowed enough time for the labelled isotopes to be incorporated into the nucleic acids of the grazers yet prevented both significant changes in the eukaryotic community, and potential indirect incorporation of labelled isotopes that could occur during an extended incubation. All water samples were filtered through 0.2-μm-pore-size membranes and preserved in RNAlater at −80°C until analysis.

### DNA and RNA extraction, gradient fractionation and cDNA synthesis

RNAlater was removed by washing the filters with cold 70% ethanol. DNA was extracted following Coffroth and colleagues (1992) protocol. Filters were placed in 0.5 ml of CTAB (hexadecyltrimethyl ammonium bromide) buffer (1.4 M NaCl, 20 mM EDTA, 100 mM Tris-HCl pH 8.0, 0.2% CTAB and 0.2% 2-mercapthoethanol) and the tubes were placed in a mini-bead beater (BioSpec Products, Bartlesville, OK, USA) and vortexed for 2 min at the maximum speed (4800 r.p.m.) to re-suspend the cells. Proteinase K was added to a final concentration of 0.1 mg ml^−1^ and samples were incubated at 65°C for 1 h. An equal volume of chloroform was added, mixed and spun at 14 000 *g* for 10 min. The aqueous layer was transferred to a new tube and DNA was extracted with an equal volume of phenol : chloroform : isoamyl alcohol (25:24:1). Finally, DNA was precipitated by addition of 2 vols of cold 95% ethanol without addition of additional salt. Pellet was washed twice with 70% cold ethanol dried and re-suspended in water.

For RNA extraction filters were placed in 100 μl of 10 mM Tris-HCl pH 8.0, 4 μl of RNase inhibitor (Ambion, Austin, TX, USA) and 2 μl lysozyme (50 mg ml^−1^). Samples were incubated for 30 min at 37°C. An additional 2 μl of the 50 mg ml^−1^ lysozyme solution was added and the samples were incubated again for 30 min at 37°C. Total RNA was immediately extracted by a mirVana RNA isolation kit (Ambion, Austin, TX, USA).

Labelled and unlabelled RNA were separated by density gradient centrifugation, performed according to the protocol of Lueders and colleagues (2004). Centrifugation media were prepared by mixing 4.5 ml of a 2 g ml^−1^ CsTFA stock solution (Amersham Pharmacia Biotech), up to 1 ml of gradient buffer (GB; 0.1 M Tris-HCl pH 8; 0.1 M KCl; 1 mM EDTA) and RNA extracts (up to 500 ng). Additionally, 175 μl of formamide was added to centrifugation media to guarantee that RNA was denatured. The average density of all prepared gradients was checked with an AR200 digital refractometer (Leica Microsystems), and adjusted by adding small volumes of Cs salt solution or gradient buffer, if necessary. 18S rRNA was resolved in CsTFA gradients with an average density of 1.8316 g ml^−1^ at 20°C. Quick-Seal Polyallomer tubes, 3.9 ml (Beckmann Instruments), were filled up with centrifugation media plus sample, and centrifuged in an Optima TLX ultracentrifuge using a TLN100 vertical rotor (Beckmann Instruments). Centrifugation conditions were > 60 h at 61 000 r.p.m. (131 000 *g*).

Centrifuged gradients were fractionated from bottom to top into 12 equal fractions (∼400 μl). A precisely controlled flow rate was achieved by displacing the gradient medium with water at the top of the tube using a syringe pump (Harvard Apparatus). The density of 15 μl from each collected fraction was determined using an AR200 digital refractometer (Leica Microsystems). Total RNA was precipitated with 1 vol. of isopropanol. Precipitates from gradient fractions were washed once with 70% ethanol and re-suspended in 25 μl of EB for subsequent determination of total RNA using RiboGreen (Molecular Probes, Invitrogen, Carlsbad, CA, USA) assays.

Primers for 18S rRNA eukaryotic genes were designed using the Design Probes tool from the ARB software (Ludwig *et al*., 2004): EukF (5′-GGGTTCGATTCCGGAGAG-3′) EukR (5′-CCGTGTTGAGTCAAATT-3′) (Integrated DNA Technologies Coralville, IA, USA). The database used contained 27 887 complete sequences, all of eukaryotic origin. EukF primer matched 19 378 sequences with 0 mismatches and 23 459 sequences with one mismatch. EukR primer matched 25 739 sequences with 0 mismatches and 27 447 sequences with one mismatch. They were tested in two cultures of *Cafeteria*, two cultures of *Paraphysomonas* and one culture of *Dullaniella*, given in all cases the expected-size PCR product of approximately 830 bp.

Total RNA (0.5–5 ng) from fractions containing highly labelled and unlabelled RNA was reverse transcribed with the specific primers using the ThermoScript RT-PCR system (Invitrogen, Carlsbad, CA, USA). Reverse transcription was performed for 2 h at 50°C.

PCR reactions were performed using Taq DNA polymerase from NEB and primers at 2 μM concentration. After 5 min at 95°C, 35 cycles of denaturation (95°C, 45 s), annealing (52°C, 1 min), elongation (72°C, 1 min) and a final elongation step (72°C, 10 min) were run in a MJ Research PTC 100 Thermal Cycler. PCR products were cleaned up using a QIAquick PCR purification kit (Qiagen, Valencia, CA, USA) and cloned into either TOPO TA cloning vector (Invitrogen, Carlsbad, CA, USA) or pGEM-T cloning vector (Promega, Madison, WI, USA). Inserts were sequenced either at Genaissance Pharmaceuticals (New Haven, CT; now Cogenics, MA, USA) using primers for the T7 promoter region or in house using the same primer and the BigDye sequencing kit (Applied Biosystems, Foster City, CA, USA) at 1 min denaturation and 25 cycles of 95°C−30 s, 50°C−20 s, 60°C−4 min, and finally held at 4°C. The reactions were then purified by ethanol precipitation and run on an ABI PRISM 3730 (Applied Biosystems) capillary DNA sequencer.

16S rRNA genes from bacteria present in the heavy fractions were cloned and sequenced using universal primers 9F (5′-GAGTTTGATYMTGGCTC) and 1509R (5′-GYTACCTTGTTACGACTT) (Integrated DNA Technologies Coralville, IA, USA). PCR and cloning were performed as described above but elongation at 72°C was extended to 2 min. Fragments were sequenced using the ABI PRISM BigDye Terminator v3.1 Cycle Sequencing Kit (Applied Biosystems, Warrington, UK) and primers for the T7 promoter region.

### Taxonomic affiliation and phylogenetic analysis

Vector contamination was assessed using VecScreen (http://www.ncbi.nlm.nih.gov/VecScreen/VecScreen.html). On the basis of the evaluation by the check_chimera program of the Ribosomal Database Project (Maidak *et al*., 2001) only sequences that showed no evidence for potential chimeric gene artefacts were analysed.

Preliminary taxonomic affiliation of the sequences was determined using blastn against the GenBank nr database (March 2005). Phylogenetic analysis was based on partial sequences trimmed to the shortest common denominator. A first analysis to confirm the taxonomic affiliation of the sequences, and have a raw picture of the overall phylogenetic tree, was performed using ARB software. Sequences were aligned against the eukaryotic database (SSRef release 90 12.05.2007, SILVA database project http://www.arb-silva.de/ with 27 887 pre-aligned sequences) (Pruesse *et al*., 2007) in the ARB software version 07.02.20 (Ludwig *et al*., 2004) and performed using the Fast Alignment tool. Alignments were edited manually and sequences were added to the backbone tree using ARB's ‘Parsimony insertion’ feature.

For maximum likelihood (ML), neighbour joining (NJ)-distance and maximum parsimony (MP) analyses, alignments were generated using MAFFT (Katoh *et al*., 2002; 2005) and edited manually using Sequence Alignment Editor v2.0 (http://tree.bio.ed.ac.uk/software/seal/). Maximum parsimony analysis was performed using the ‘fast’ stepwise-addition algorithm in paup 4.0b10 (Altivec) with 1000 bootstraps replicates. For each alignment the best DNA substitution model was evaluated using MrModeltest 2.2 (Nylander, 2004), which ranked General Time Reversible-gamma-Proportion invariant (GTR+g+I) best model in all cases. Maximum likelihood analysis was performed using the software PHYML_v2.4.4 (Guindon and Gascuel, 2003) and GTR as a substitution model with 100 bootstraps replicates. Neighbour joining-distance analysis was performed using paup 4.0b10 (Altivec) using the also GTR as a substitution model, with 1000 bootstraps replicates, and the values of Gamma-shape and proportion of invariable sites estimated by PHYML. Trees were visualized and plotted using NJPlot v2.1 (Perriere and Gouy, 1996).

### T-RFLP analysis

Fluorescently labelled PCR products for the T-RFLP analysis were generated by the PCR protocol described above, using a FAM-labelled forward primer. PCR products were digested with the restriction endonucleases HhaI and RsaI (New England Biolabs, Ipswich, MA, USA). The resulting fluorescent terminal fragments were resolved and analysed at the Roy J. Carver Biotechnology Center (University of Illinois at Urbana-Champaign) using an ABI Prism 3730xl Analyser automated sequencer, and GeneMapper version 3.7 software.

Clustering of the different T-RFLP profiles was performed using the Self-Organizing Tree Algorithm (SOTA) from the GEPAS 4.0 (GEPAS website http://www.gepas.org).

### Chloroplast 16S rRNA analysis

Labelled fractions from both *Prochlorococcus* and *Synechococcus* grazers were tested for the presence of 16S rRNA chloroplast sequences. Specific oligonucleotides against chloroplast sequences (SSRef release 90 12.05. 2007, SILVA database project http://www.arb-silva.de/) were design using the Design Probes tool from the ARB software (Ludwig *et al*., 2004). Although a total of 16 sets of primers were used in the experiment (Table S1), only the set of primers 15F (5′-TTAACTCAAGTG GCGGACGG) and 15R (AGTGTTAG TAATAGCCCAGTA) gave a PCR product long enough to be sequenced. PCR reactions were performed using Taq DNA polymerase from NEB and primers at 2 μM concentration. After 5 min at 95°C, 40 cycles of denaturation (95°C, 45 s), annealing (56°C, 1 min), elongation (72°C, 1 min) and a final elongation step (72°C, 10 min) were run in a MJ Research PTC 100 Thermal Cycler. PCR products were clean up using a QIAquick PCR purification kit (Qiagen, Valencia, CA, USA) and cloned into TOPO TA cloning vector (Invitrogen, Carlsbad, CA, USA) and sequenced as described above.

### Nucleotide sequence accession numbers

Ribosomal RNA sequences have been deposited at GenBank/EMBL under Accession Nos EF695076–EF695247 and EU499951–EU500232.

## Supporting information

**Fig. S1.** Phylogenetic analysis of the sequences derived from the 18S rDNA sequences in the indigenous microbial community. Unrooted phylogenetic tree inferred by maximum likelihood (ML) analysis. A total of 1090 positions used, including gaps (sequences ranging from a minimum of 585 bp up to 893 bp), from an alignment of 203 partial sequences were used. Bootstrap values over 50% are indicated on the internal branches obtained from both ML, neighbour joining-distance methods (NJ-Dis) and using maximum parsimony (MP) (in the order ML/NJ-Dist/MP). Bootstrap values < 50%, or not supported at least in two of the analyses, have been omitted. The gamma distribution parameter (α) was estimated at 0.520; and the proportion of invariant sites (*I*) was 0.015. The scale bar indicates 10% divergence. The sequences from the duplicate biological samples are indicated as (dhot1) and (dhot2). Classification is based on Adl and colleagues (2005) and Not and colleagues (2007). All groups correspond to first and second rank according to Adl and colleagues (2005) except *Super-group and **Phylum (Shalchian-Tabrizi *et al*., 2006).

**Fig. S2.** Phylogenetic analysis of the sequences derived from the 18S rDNA sequences in the unlabelled fractions from the experiments (time 0 and blue circles in Fig. 2). Unrooted SSU rRNA-derived library phylogenetic tree of eukaryotes inferred by maximum likelihood (ML) analysis. A total of 1058 positions used, including gaps (sequences ranging from a minimum of 512 bp up to 886 bp), from an alignment of 200 partial sequences were used. Bootstrap values over 50% are indicated on the internal branches obtained from both ML, neighbour joining-distance methods (NJ-Dis) and using maximum parsimony (MP) (in the order ML/NJ-Dist/MP). Bootstrap values < 50%, or not supported at least in two of the analyses, have been omitted. The gamma distribution parameter (α) was estimated at 0.594; and the proportion of invariant sites (*I*) was 0.000. The scale bar indicates 10% divergence. The sequences coming from the duplicate biological samples is indicated as (A) and (B). Clones colour code: dark red: sequences from the time 0 sample, representing the metabolically active initial eukaryotic microbial community; purple: sequences from the unlabelled eukaryotic RNA obtained from the samples incubated with *Prochlorococcus*; orange: sequences from the unlabelled fraction from the samples incubated with *Synechococcus*. Classification is based on Adl and colleagues (2005) and Not and colleagues (2007). All groups correspond to first and second rank according to Adl and colleagues (2005) except when noted as follows: *Super-group and **Phylum (Shalchian-Tabrizi *et al*., 2006). ***Unidentified chloroplastida, BLAST results gave no clear match and the sequences did not cluster clearly with any of the second-rank groups used in the tree that could indicate the exact affiliation of the sequence.

**Fig. S3.** Phylogenetic analysis of the sequences derived from the labelled 18S rDNA sequences (red circles in Fig. 2) from the experimental bottles amended with labelled *Prochlorococcus* cells. Unrooted 18S rRNA-derived library phylogenetic tree of eukaryotes inferred by maximum likelihood (ML) analysis. A total of 1145 positions used, including gaps (sequences ranging from a minimum of 545 bp up to 980 bp), from an alignment of 192 partial sequences were used. Bootstrap values over 50% are indicated on the internal branches obtained from both ML, neighbour joining-distance methods (NJ-Dis) and using maximum parsimony (MP) (in the order ML/NJ-Dist/MP). Bootstrap values < 50%, or not supported at least in two of the analyses, have been omitted. The gamma distribution parameter (α) was estimated at 0.512; and the proportion of invariant sites (*I*) was 0.000. The scale bar indicates 10% divergence. The sequences coming from the duplicate biological samples is indicated as (A) and (B). Classification is based on Adl and colleagues (2005) and Not and colleagues (2007). All groups correspond to first and second rank according to Adl and colleagues (2005) except when noted as follows: *Super-group and **Phylum (Shalchian-Tabrizi *et al*., 2006). ***Unidentified stramenopiles, blast results gave no clear match and the sequences did not cluster clearly with any of the second-rank groups used in the tree that could indicate the exact affiliation of the sequence.

**Fig. S4.** Phylogenetic analysis of the sequences derived from the labelled 18S rDNA sequences (red circles in Fig. 2) from the experimental bottles amended with labelled *Synechococcus* cells. Unrooted 18S rRNA-derived library phylogenetic tree of eukaryotes inferred by maximum likelihood (ML) analysis. A total of 1156 positions used, including gaps (sequences ranging from a minimum of 507 bp up to 977 bp), from an alignment of 188 partial sequences were used. Bootstrap values over 50% are indicated on the internal branches obtained from both ML, neighbour joining-distance methods (NJ-Dis) and using maximum parsimony (MP) (in the order ML/NJ-Dist/MP). Bootstrap values < 50%, or not supported at least in two of the analyses, have been omitted. The gamma distribution parameter (α) was estimated at 0.543; and the proportion of invariant sites (*I*) was 0.036. The scale bar indicates 10% divergence. The sequences coming from the duplicate biological samples is indicated as (A) and (B). Classification is based on Adl and colleagues (2005) and Not and colleagues (2007). All groups correspond to first and second rank according to Adl and colleagues (2005) except when noted as follows: *Super-group and **Phylum (Shalchian-Tabrizi *et al*., 2006). ***Unidentified stramenopiles, BLAST results gave no clear match and the sequences did not cluster clearly with any of the second-rank groups used in the tree that could indicate the exact affiliation of the sequence.

**Fig. S5.** Phylogenetic tree 16S rRNA sequences from chloroplasts and bacteria inferred by maximum likelihood (ML) analysis. Blue: cyanobacterial 16S rRNA sequences. Green: sequences originating from the labelled fraction of the *Prochlorococcus* inoculation experiment. Orange: sequences originated from the labelled fraction from the *Synechococcus* inoculation experiment. (A) and (B) represent the two biological replicates in the experiments. A total of 724 positions were used, including gaps (sequences ranging from a minimum of 358 bp up to 668 bp), from an alignment of 103 partial sequences. Bootstrap values over 50% are indicated on the internal branches obtained from ML, neighbour joiningdistance methods (NJ-Dist) and using maximum parsimony (MP) (in the order ML/NJ-Dist/MP). Bootstrap values < 50%, or not supported at least in two of the analyses, have been omitted. The proportion of invariant sites (*I*) was 0.241. The scale bar indicates 10% divergence. An archaeal sequence was used as out-group (*Sulfolobus acidocaldarius*).

**Table S1.** Oligonucleotides used for the amplification of 16S rRNA chloroplast genes from different groups defined based on the ARB tree (SSRef release 90 12.05.2007) for these group of sequences. F, forward primer. R, reverse primer.
