# Supplementary material for: Use of stable isotope-labelled cells to identify active grazers of picocyanobacteria in ocean surface waters
Source: Environ Microbiol. 2009 Feb;11(2):512–25. doi: 10.1111/j.1462-2920.2008.01793.x (PMC2702499; doi:10.1111/j.1462-2920.2008.01793.x)
Supplement: Supplementary file 1 [file emi0011-0512-SD1.pdf]

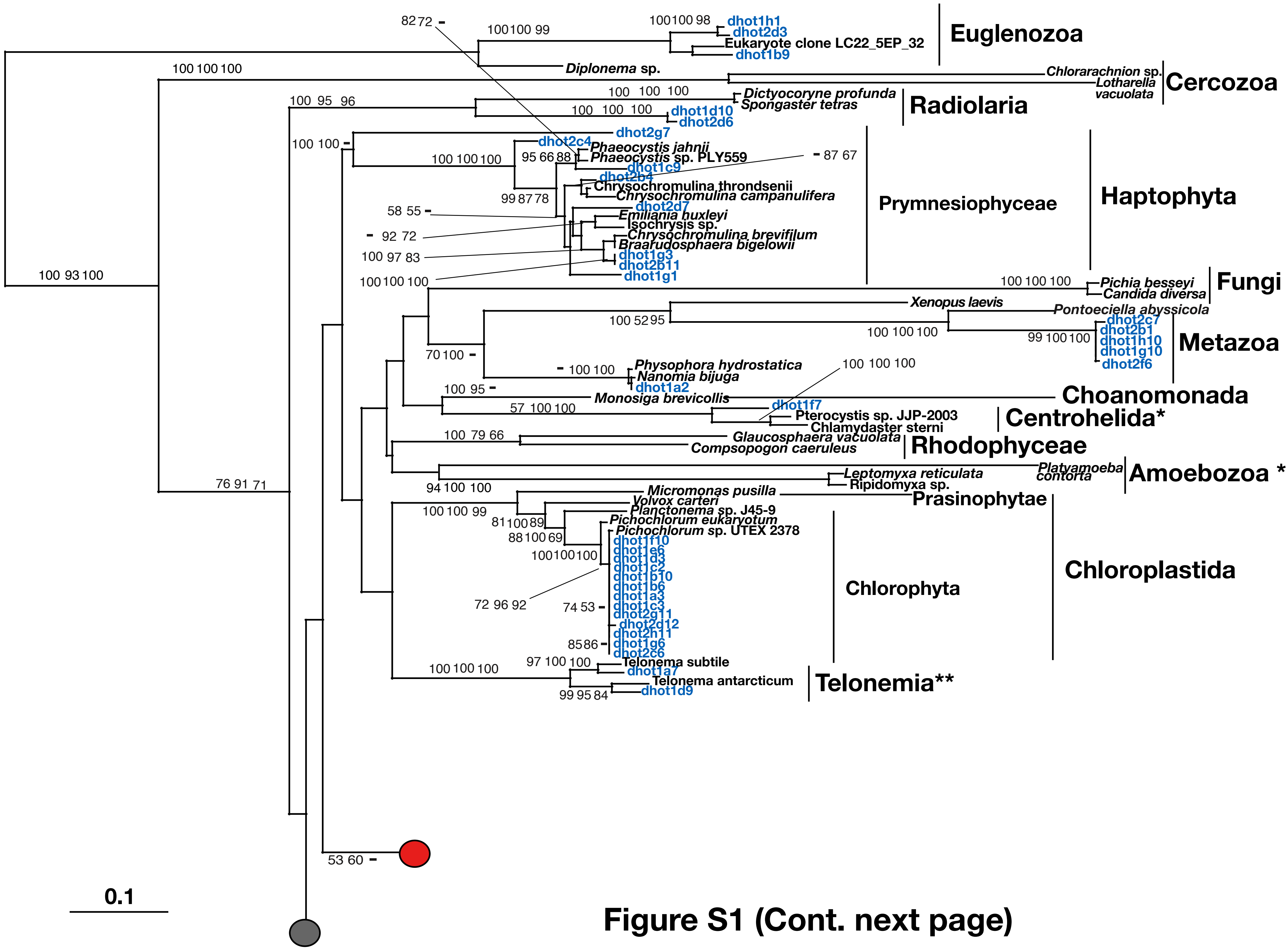

Figure S1 (Cont. next page)

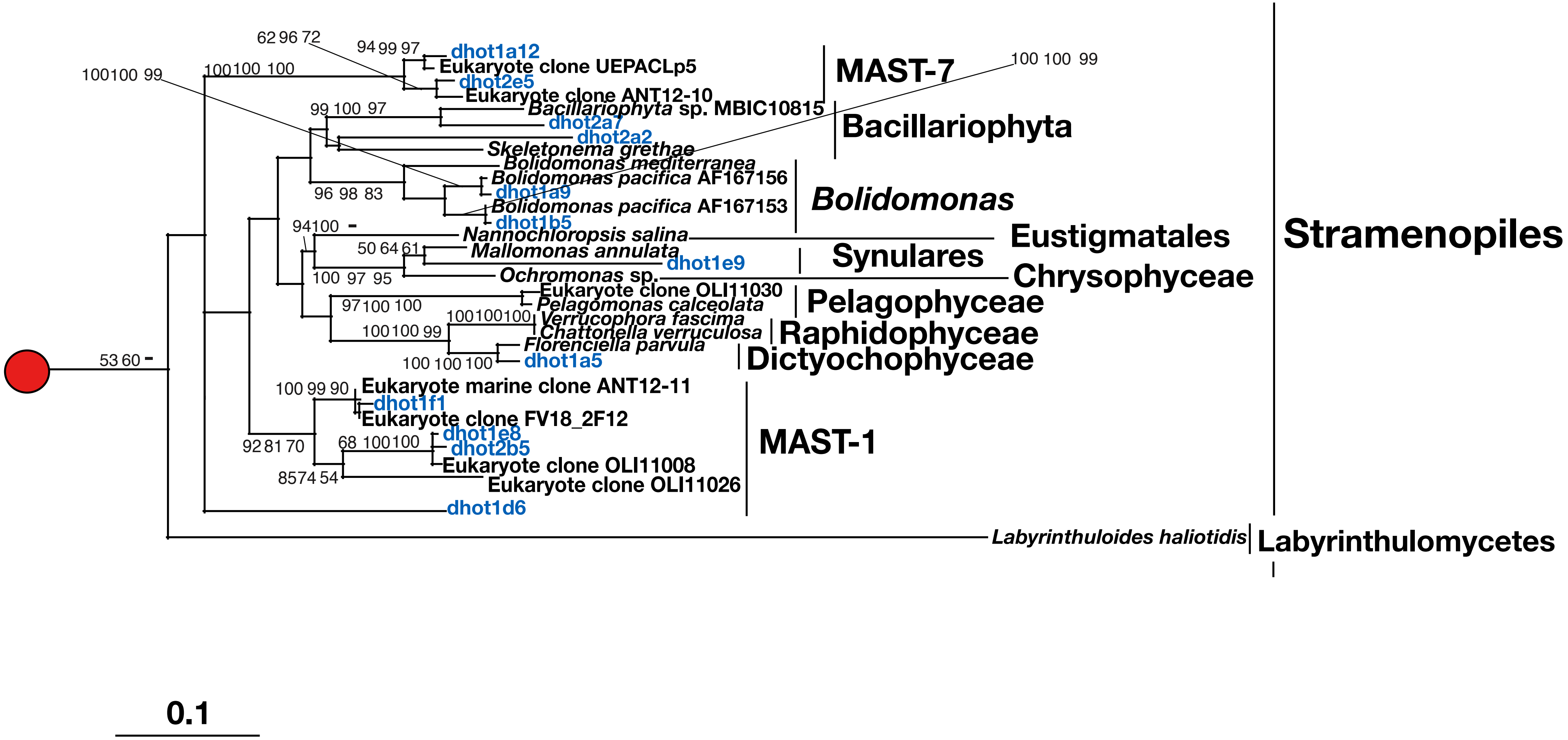

Figure S1 (Cont. next page)

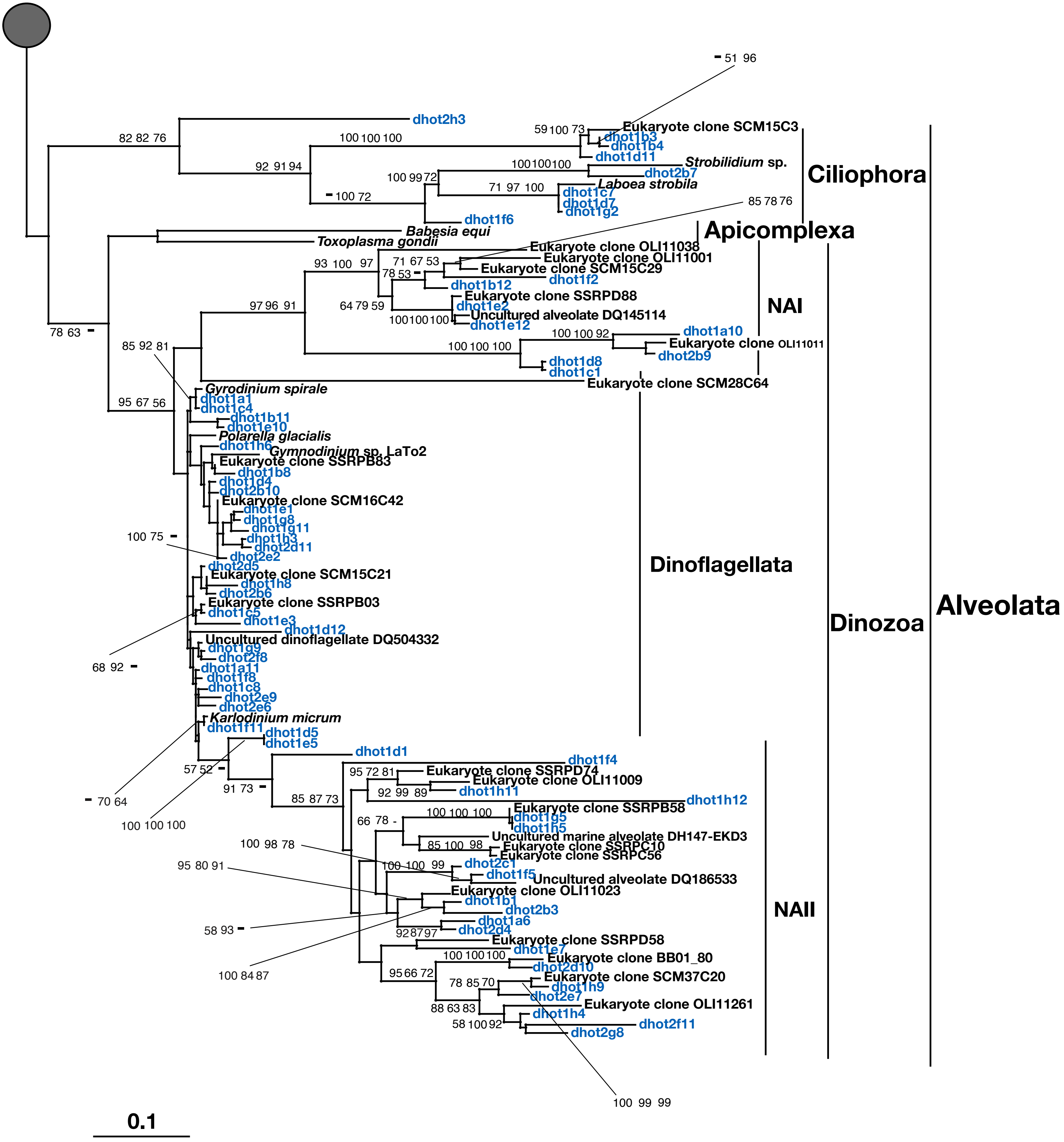

Ciliophora
Apicomplexa
NAI
Dinoflagellata
Dinozoa
Alveolata
NAII
0.1
dhot2h3
Eukaryote clone SCM15C3
Strobilidium sp.
Laboea strobila
Babesia equi
Toxoplasma gondii
Eukaryote clone OLI11038
Eukaryote clone OLI11001
Eukaryote clone SCM15C29
Eukaryote clone SSRPD88
Uncultured alveolate DQ145114
Eukaryote clone OLI11011
Eukaryote clone SCM28C64
Gyrodinium spirale
Polarella glacialis
Gymnodinium sp. LaTo2
Eukaryote clone SSRPB83
Eukaryote clone SCM16C42
Eukaryote clone SCM15C21
Eukaryote clone SSRPB03
Uncultured dinoflagellate DQ504332
Karlodinium micrum
Eukaryote clone SSRPD74
Eukaryote clone OLI11009
Eukaryote clone SSRPB58
Uncultured marine alveolate DH147-EKD3
Eukaryote clone SSRPC10
Eukaryote clone SSRPC56
Uncultured alveolate DQ186533
Eukaryote clone OLI11023
Eukaryote clone SSRPD58
Eukaryote clone BB01_80
Eukaryote clone SCM37C20
Eukaryote clone OLI11261

Figure S1

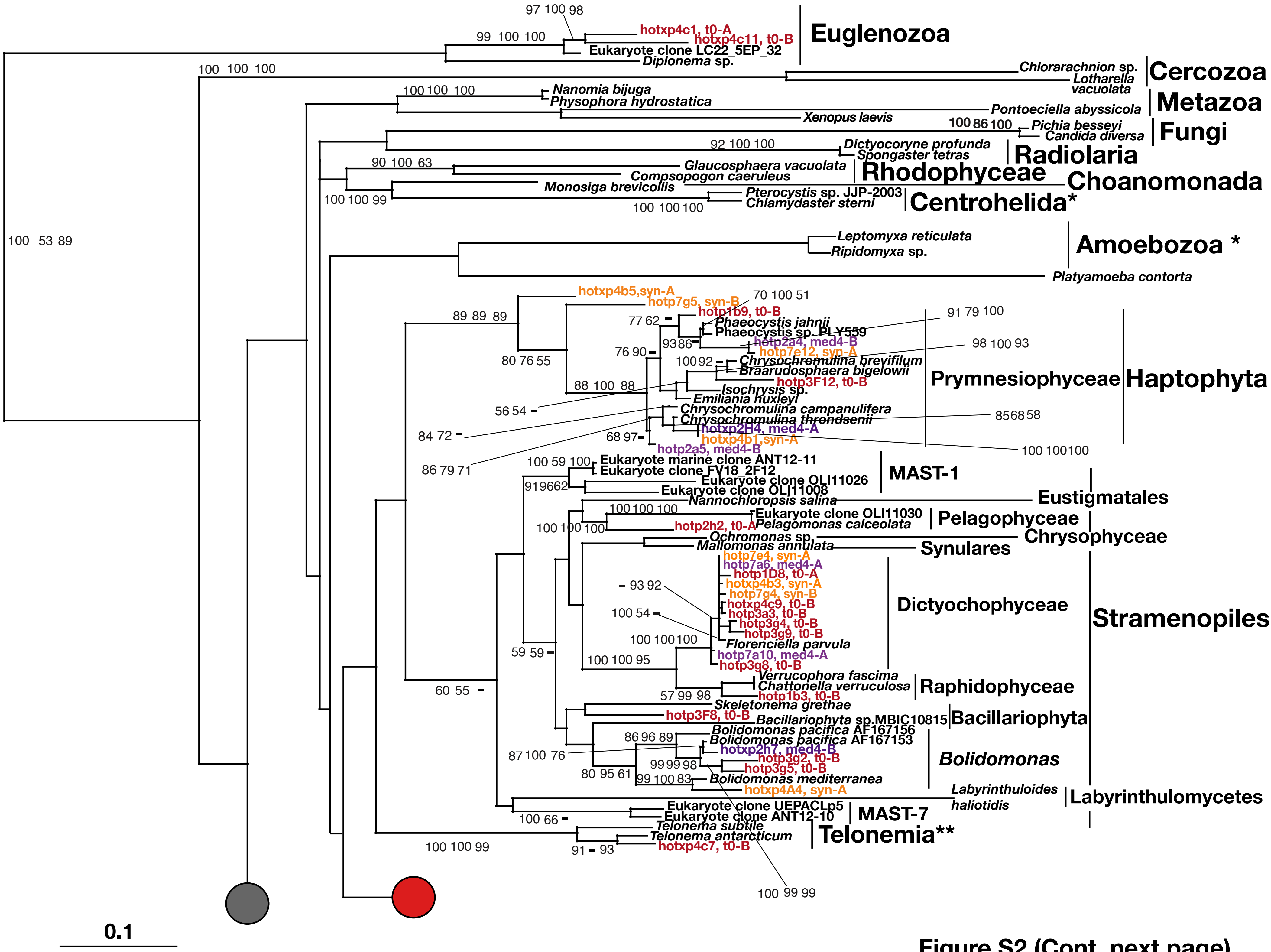

Figure S2 (Cont. next page)

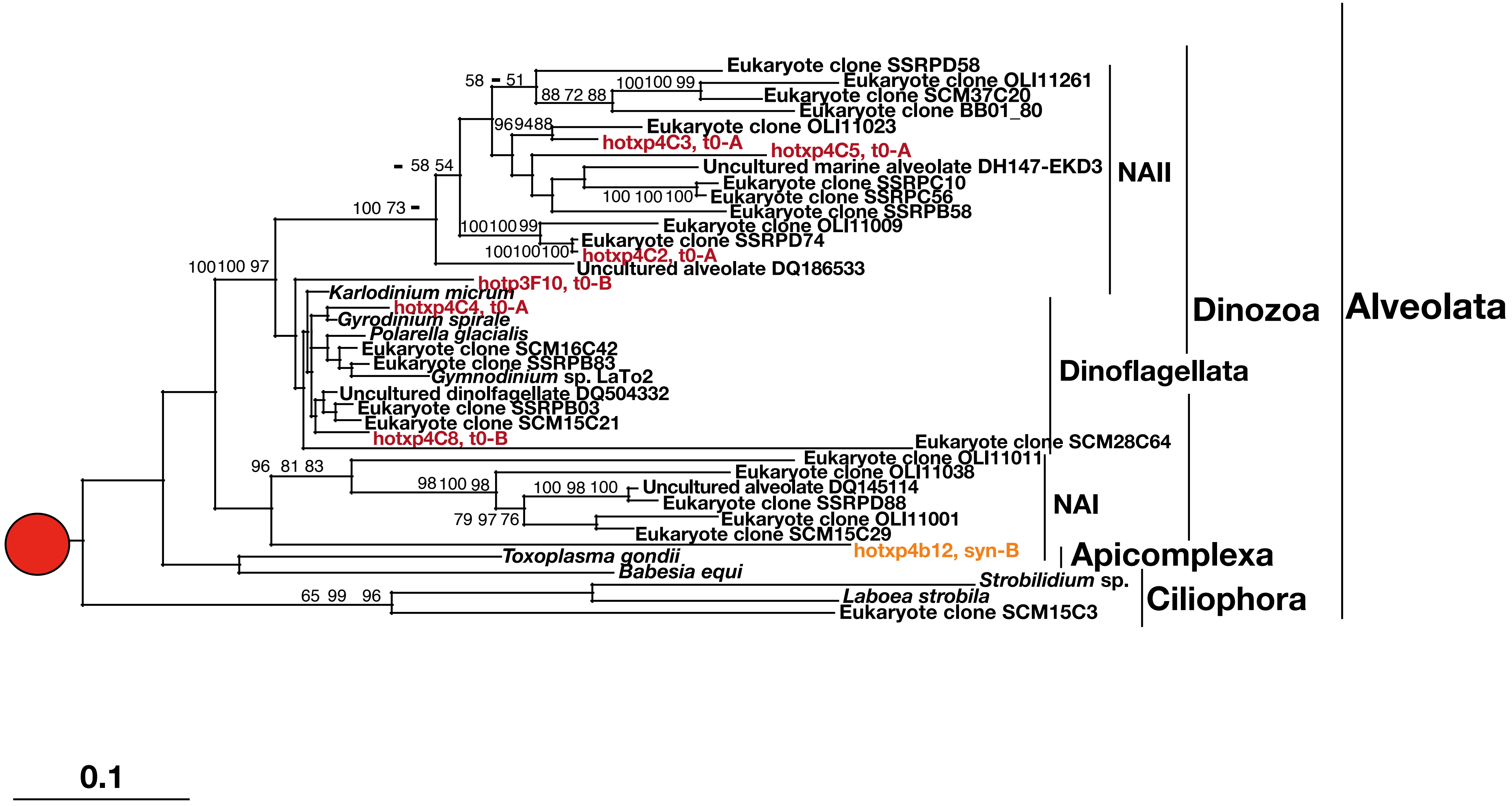

Figure S2 (Cont. next page)

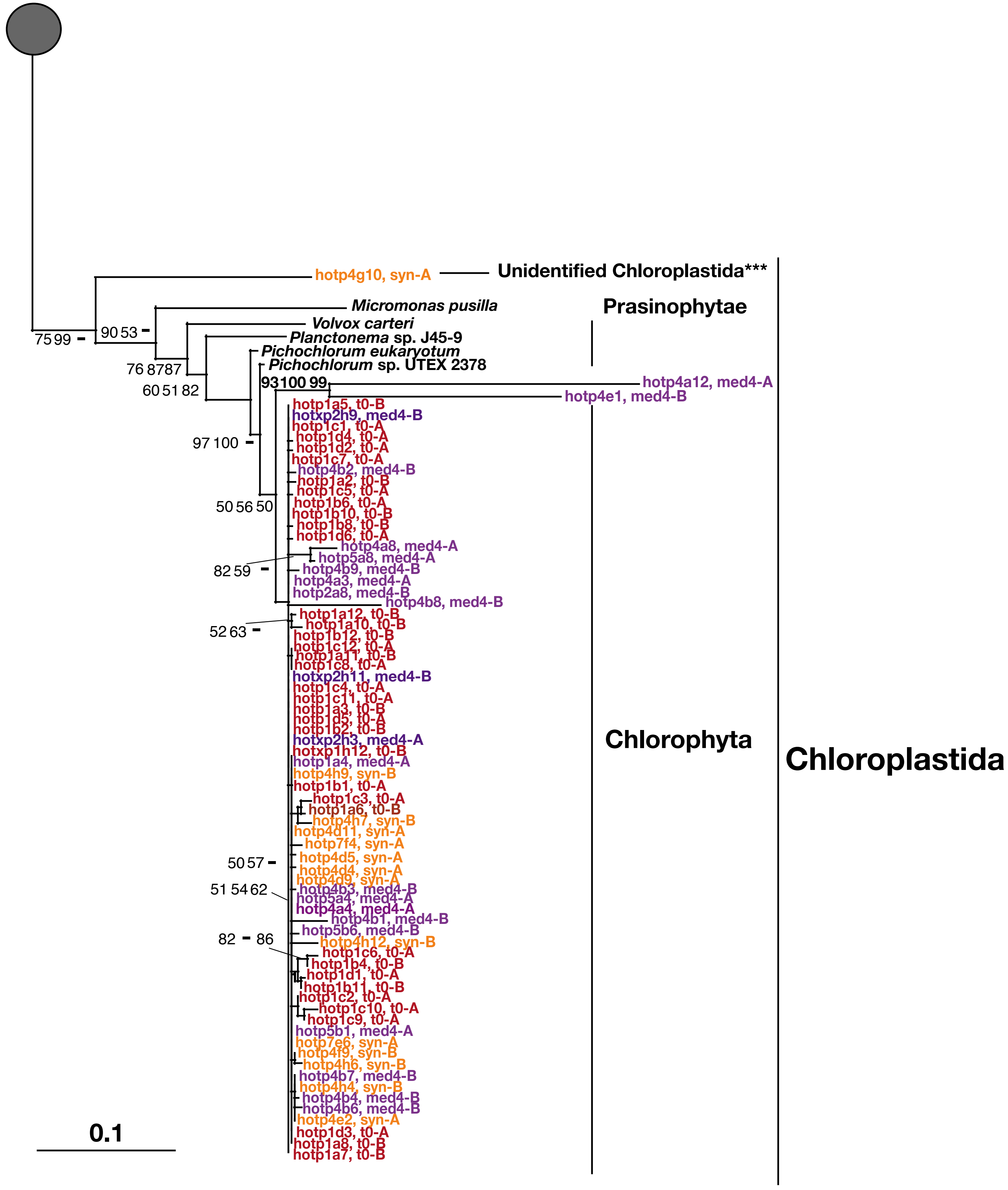
Unidentified Chloroplastida***
hotp4g10, syn-A
Prasinophytae
Micromonas pusilla
Volvox carteri
Planctonema sp. J45-9
Pichochlorum eukaryotum
Pichochlorum sp. UTEX 2378
75 99 -
90 53 -
76 87 87
60 51 82
93 100 99
hotp4a12, med4-A
hotp4e1, med4-B
97 100 -
50 56 50
82 59 -
52 63 -
50 57 -
51 54 62
82 - 86
hotp1a5, t0-B
hotxp2h9, med4-B
hotp1c1, t0-A
hotp1d4, t0-A
hotp1d2, t0-A
hotp1c7, t0-A
hotp4b2, med4-B
hotp1a2, t0-B
hotp1c5, t0-A
hotp1b6, t0-A
hotp1b10, t0-B
hotp1b8, t0-B
hotp1d6, t0-A
hotp4a8, med4-A
hotp5a8, med4-A
hotp4b9, med4-B
hotp4a3, med4-A
hotp2a8, med4-B
hotp4b8, med4-B
hotp1a12, t0-B
hotp1a10, t0-B
hotp1b12, t0-B
hotp1c12, t0-A
hotp1a11, t0-B
hotp1c8, t0-A
hotxp2h11, med4-B
hotp1c4, t0-A
hotp1c11, t0-A
hotp1a3, t0-B
hotp1d5, t0-A
hotp1b2, t0-B
hotxp2h3, med4-A
hotxp1h12, t0-B
hotp1a4, med4-A
hotp4h9, syn-B
hotp1b1, t0-A
hotp1c3, t0-A
hotp1a6, t0-B
hotp4h7, syn-B
hotp4d11, syn-A
hotp7f4, syn-A
hotp4d5, syn-A
hotp4d4, syn-A
hotp4d9, syn-A
hotp4b3, med4-B
hotp5a4, med4-A
hotp4a4, med4-A
hotp4b1, med4-B
hotp5b6, med4-B
hotp4h12, syn-B
hotp1c6, t0-A
hotp1b4, t0-B
hotp1d1, t0-A
hotp1b11, t0-B
hotp1c2, t0-A
hotp1c10, t0-A
hotp1c9, t0-A
hotp5b1, med4-A
hotp7e6, syn-A
hotp4f9, syn-B
hotp4h6, syn-B
hotp4b7, med4-B
hotp4h4, syn-B
hotp4b4, med4-B
hotp4b6, med4-B
hotp4e2, syn-A
hotp1d3, t0-A
hotp1a8, t0-B
hotp1a7, t0-B
Chlorophyta
Chloroplastida
0.1

Figure S2

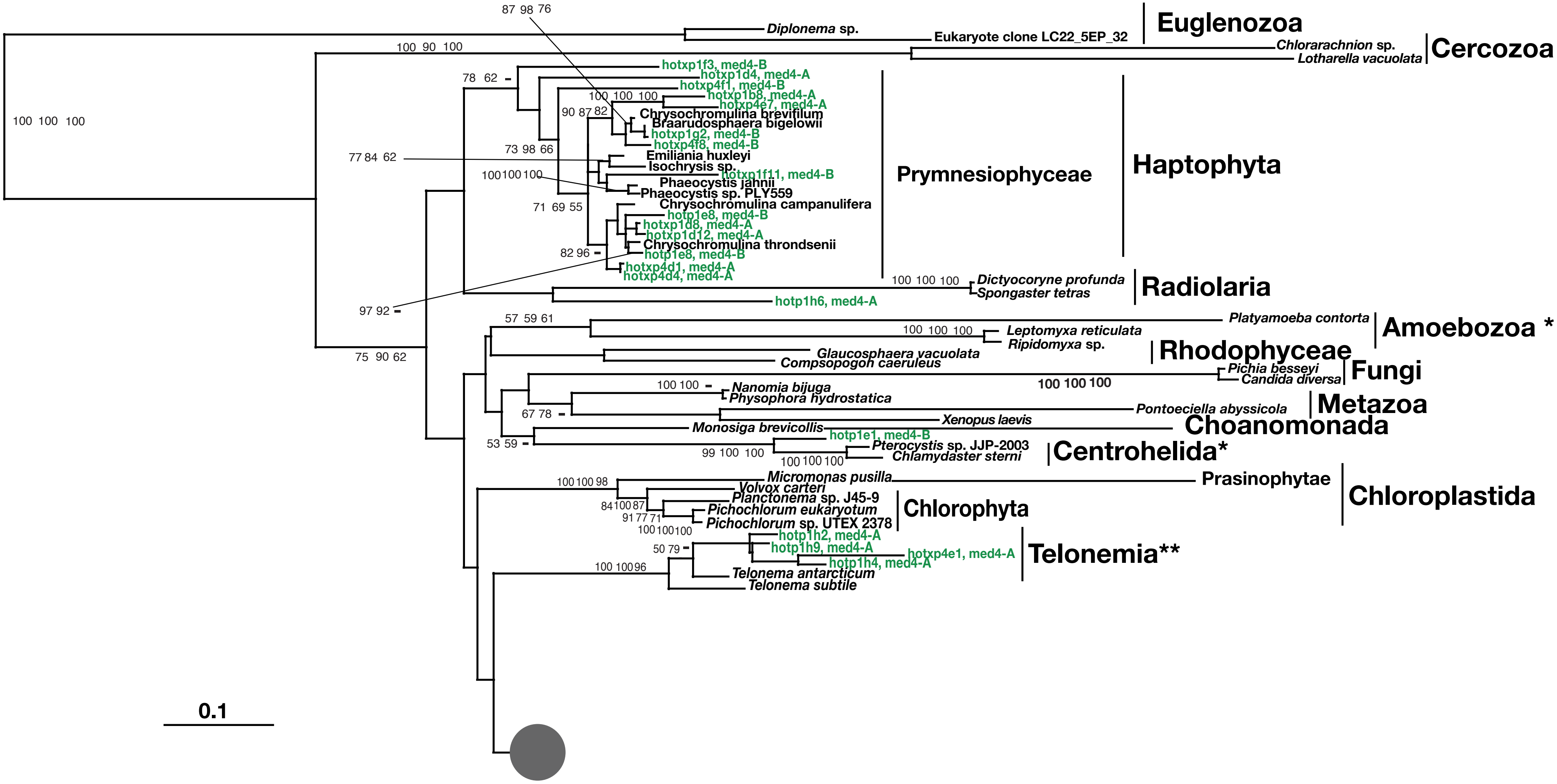

Figure S3 (Cont. next page)

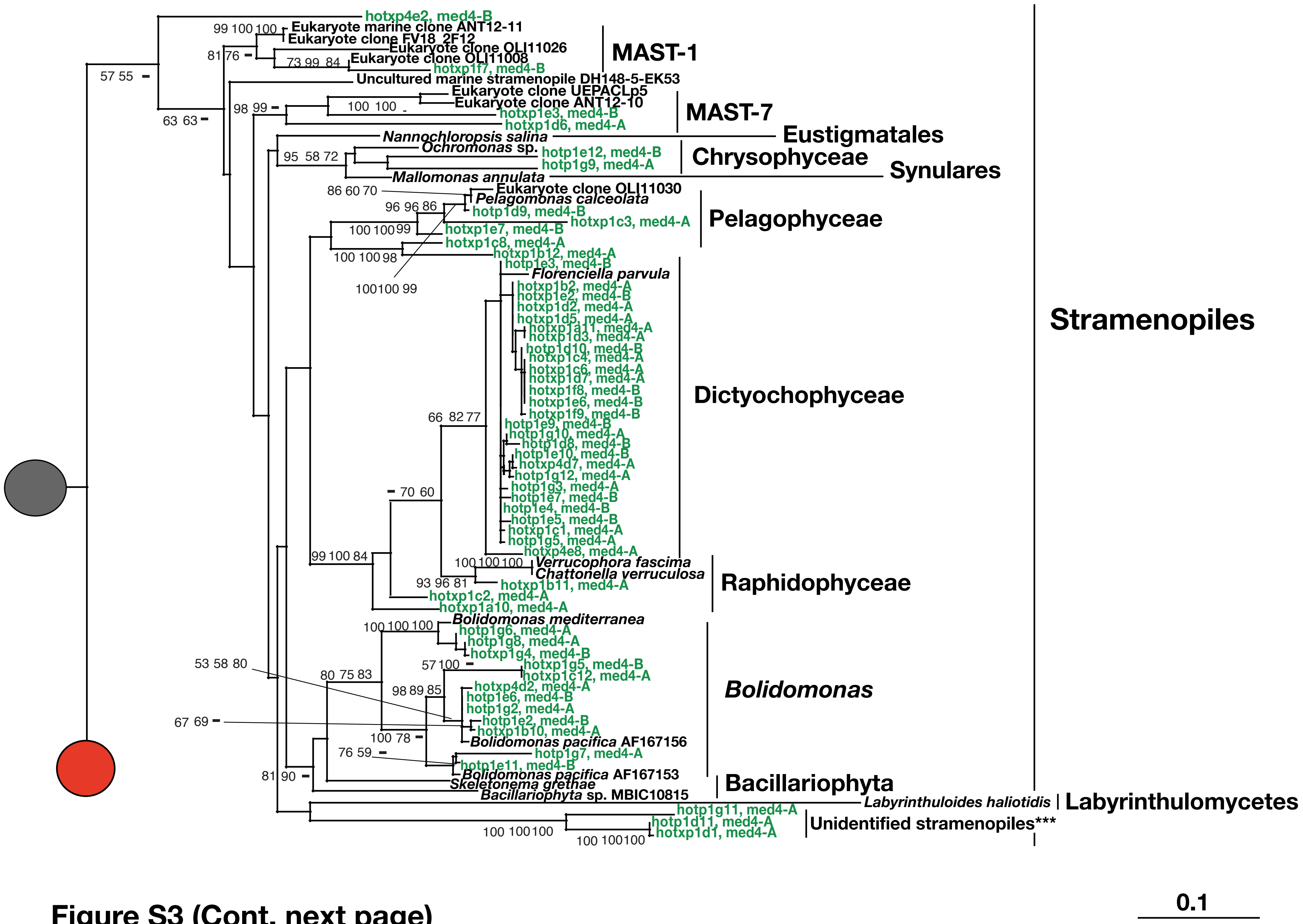

Figure S3 (Cont. next page)

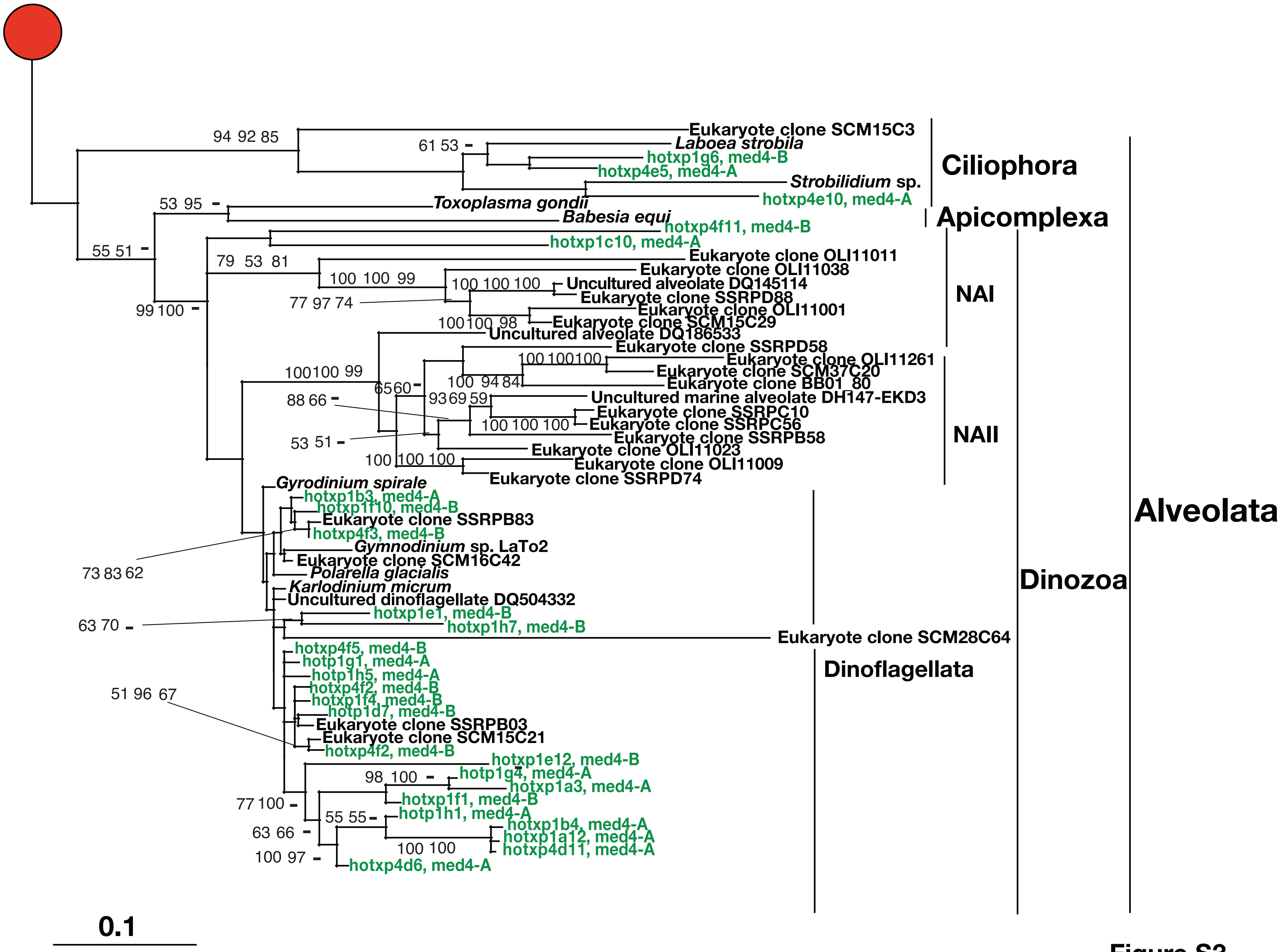
Eukaryote clone SCM15C3
Laboea strobila
hotxp1g6, med4-B
hotxp4e5, med4-A
Strobilidium sp.
hotxp4e10, med4-A
Toxoplasma gondii
Babesia equi
hotxp4f11, med4-B
hotxp1c10, med4-A
Eukaryote clone OLI11011
Eukaryote clone OLI11038
Uncultured alveolate DQ145114
Eukaryote clone SSRPD88
Eukaryote clone OLI11001
Eukaryote clone SCM15C29
Uncultured alveolate DQ186533
Eukaryote clone SSRPD58
Eukaryote clone OLI11261
Eukaryote clone SCM37C20
Eukaryote clone BB01_80
Uncultured marine alveolate DH147-EKD3
Eukaryote clone SSRPC10
Eukaryote clone SSRPC56
Eukaryote clone SSRPB58
Eukaryote clone OLI11023
Eukaryote clone OLI11009
Eukaryote clone SSRPD74
Gyrodinium spirale
hotxp1b3, med4-A
hotxp1f10, med4-B
Eukaryote clone SSRPB83
hotxp4f3, med4-B
Gymnodinium sp. LaTo2
Eukaryote clone SCM16C42
Polarella glacialis
Karlodinium micrum
Uncultured dinoflagellate DQ504332
hotxp1e1, med4-B
hotxp1h7, med4-B
Eukaryote clone SCM28C64
hotxp4f5, med4-B
hotp1g1, med4-A
hotp1h5, med4-A
hotxp4f2, med4-B
hotxp1f4, med4-B
hotp1d7, med4-B
Eukaryote clone SSRPB03
Eukaryote clone SCM15C21
hotxp4f2, med4-B
hotxp1e12, med4-B
hotp1g4, med4-A
hotxp1a3, med4-A
hotxp1f1, med4-B
hotp1h1, med4-A
hotxp1b4, med4-A
hotxp1a12, med4-A
hotxp4d11, med4-A
hotxp4d6, med4-A
94 92 85
61 53 -
53 95 -
55 51 -
79 53 81
100 100 99
100 100 100
77 97 74
99 100 -
100 100 98
100 100 99
100 100 100
100 94 84
65 60 -
93 69 59
88 66 -
100 100 100
53 51 -
100 100 100
73 83 62
63 70 -
51 96 67
98 100 -
77 100 -
55 55 -
63 66 -
100 100
100 97 -
Ciliophora
Apicomplexa
NAI
NAII
Dinozoa
Alveolata
Dinoflagellata
0.1

Figure S3

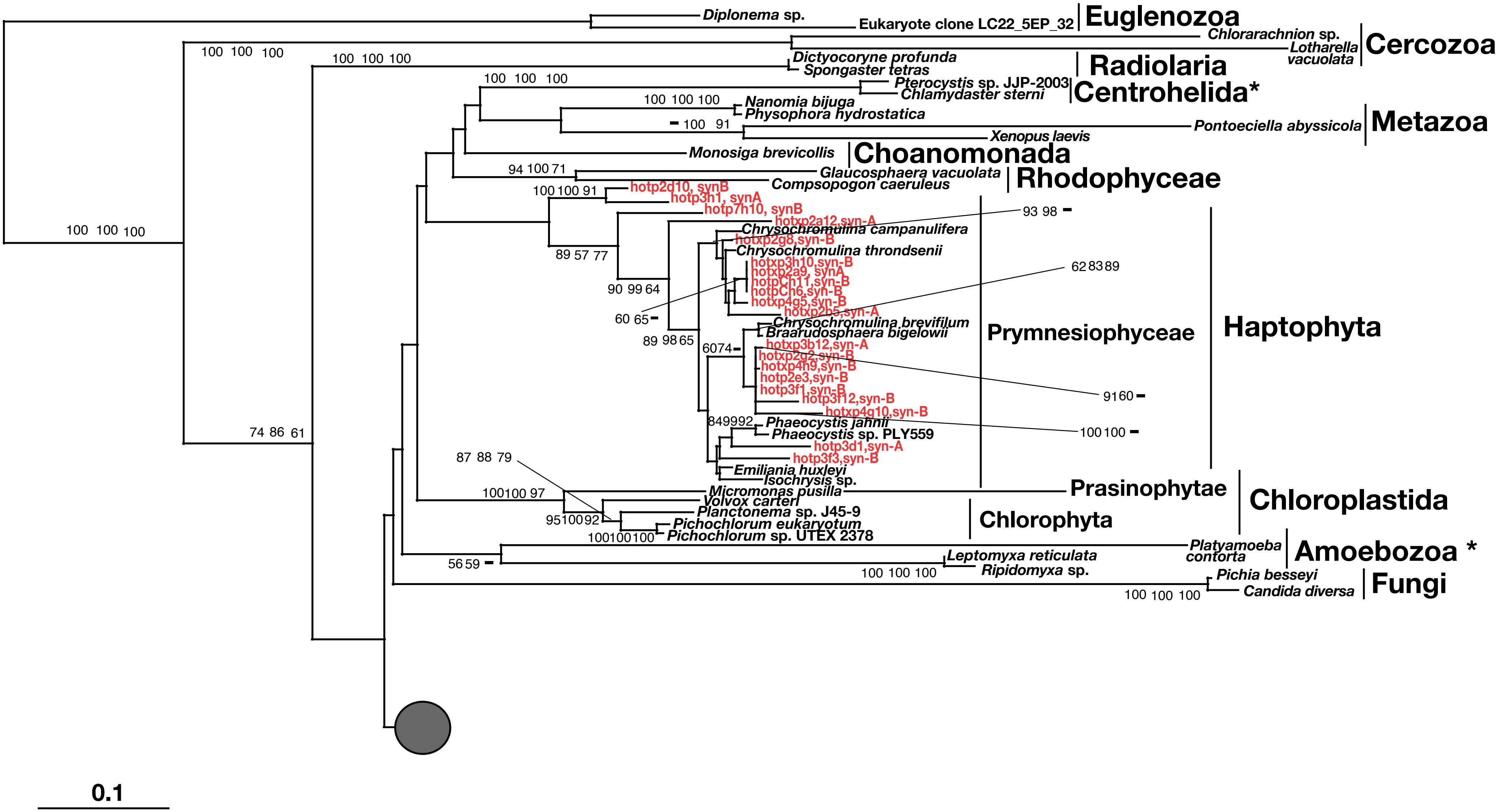

Figure S4 (Cont. next page)

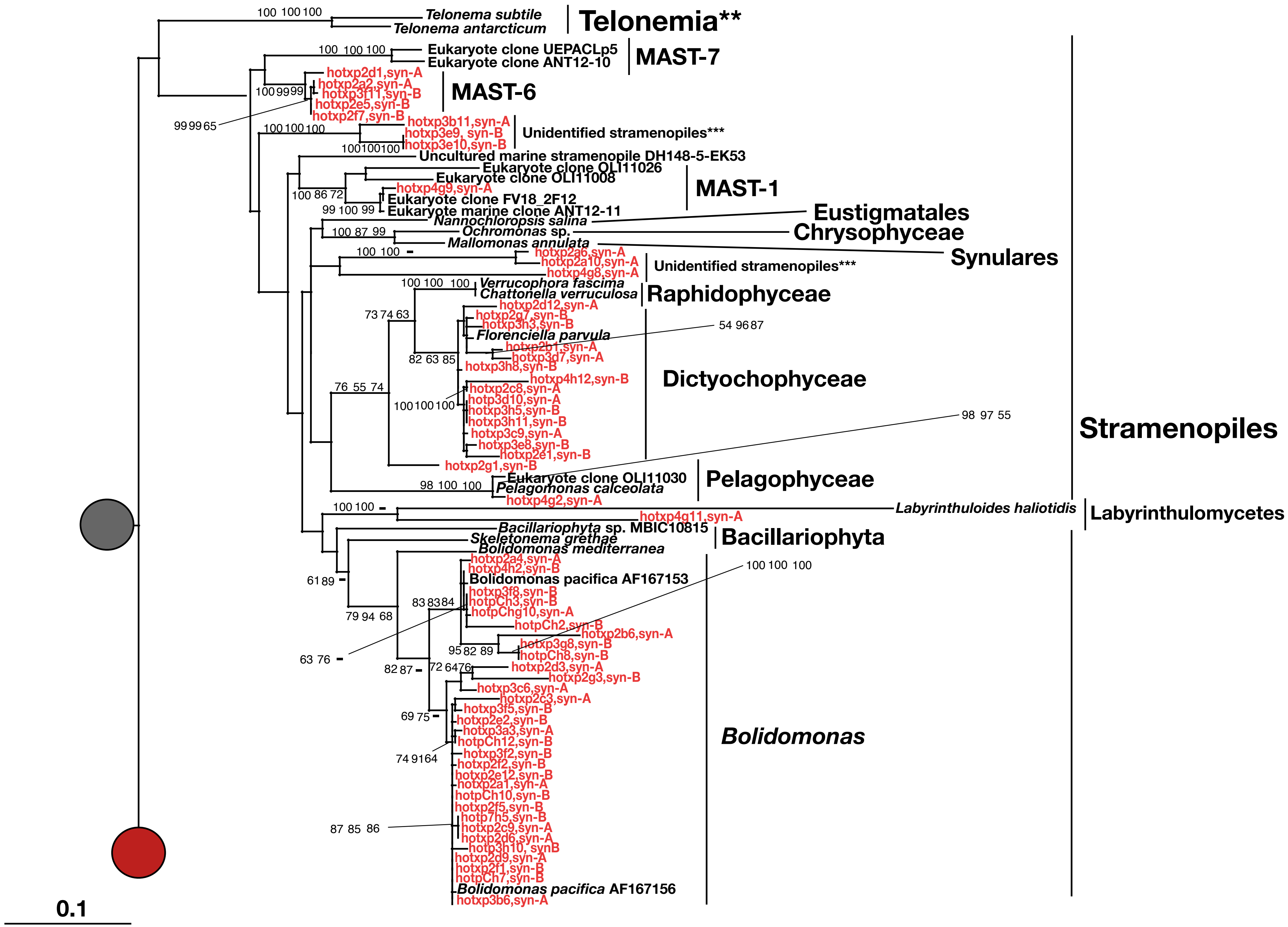

Figure S4 (Cont. next page)

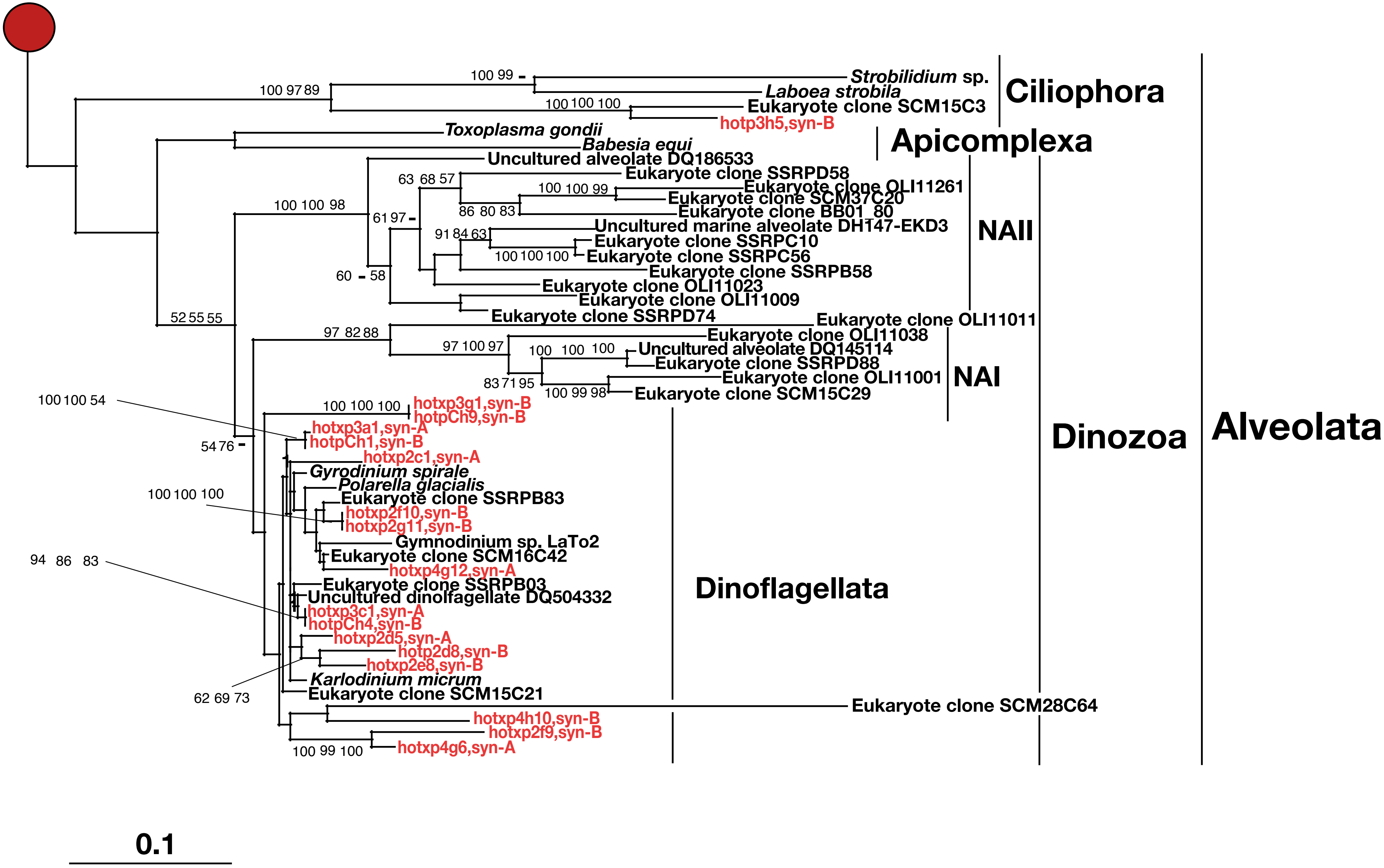

Strobilidium sp.
Laboea strobila
Eukaryote clone SCM15C3
hotp3h5,syn-B
Toxoplasma gondii
Babesia equi
Uncultured alveolate DQ186533
Eukaryote clone SSRPD58
Eukaryote clone OLI11261
Eukaryote clone SCM37C20
Eukaryote clone BB01_80
Uncultured marine alveolate DH147-EKD3
Eukaryote clone SSRPC10
Eukaryote clone SSRPC56
Eukaryote clone SSRPB58
Eukaryote clone OLI11023
Eukaryote clone OLI11009
Eukaryote clone SSRPD74
Eukaryote clone OLI11011
Eukaryote clone OLI11038
Uncultured alveolate DQ145114
Eukaryote clone SSRPD88
Eukaryote clone OLI11001
Eukaryote clone SCM15C29
hotxp3g1,syn-B
hotpCh9,syn-B
hotxp3a1,syn-A
hotpCh1,syn-B
hotxp2c1,syn-A
Gyrodinium spirale
Polarella glacialis
Eukaryote clone SSRPB83
hotxp2f10,syn-B
hotxp2g11,syn-B
Gymnodinium sp. LaTo2
Eukaryote clone SCM16C42
hotxp4g12,syn-A
Eukaryote clone SSRPB03
Uncultured dinolfagellate DQ504332
hotxp3c1,syn-A
hotpCh4,syn-B
hotxp2d5,syn-A
hotp2d8,syn-B
hotxp2e8,syn-B
Karlodinium micrum
Eukaryote clone SCM15C21
Eukaryote clone SCM28C64
hotxp4h10,syn-B
hotxp2f9,syn-B
hotxp4g6,syn-A
Ciliophora
Apicomplexa
NAII
NAI
Dinoflagellata
Dinozoa
Alveolata
0.1

Figure S4

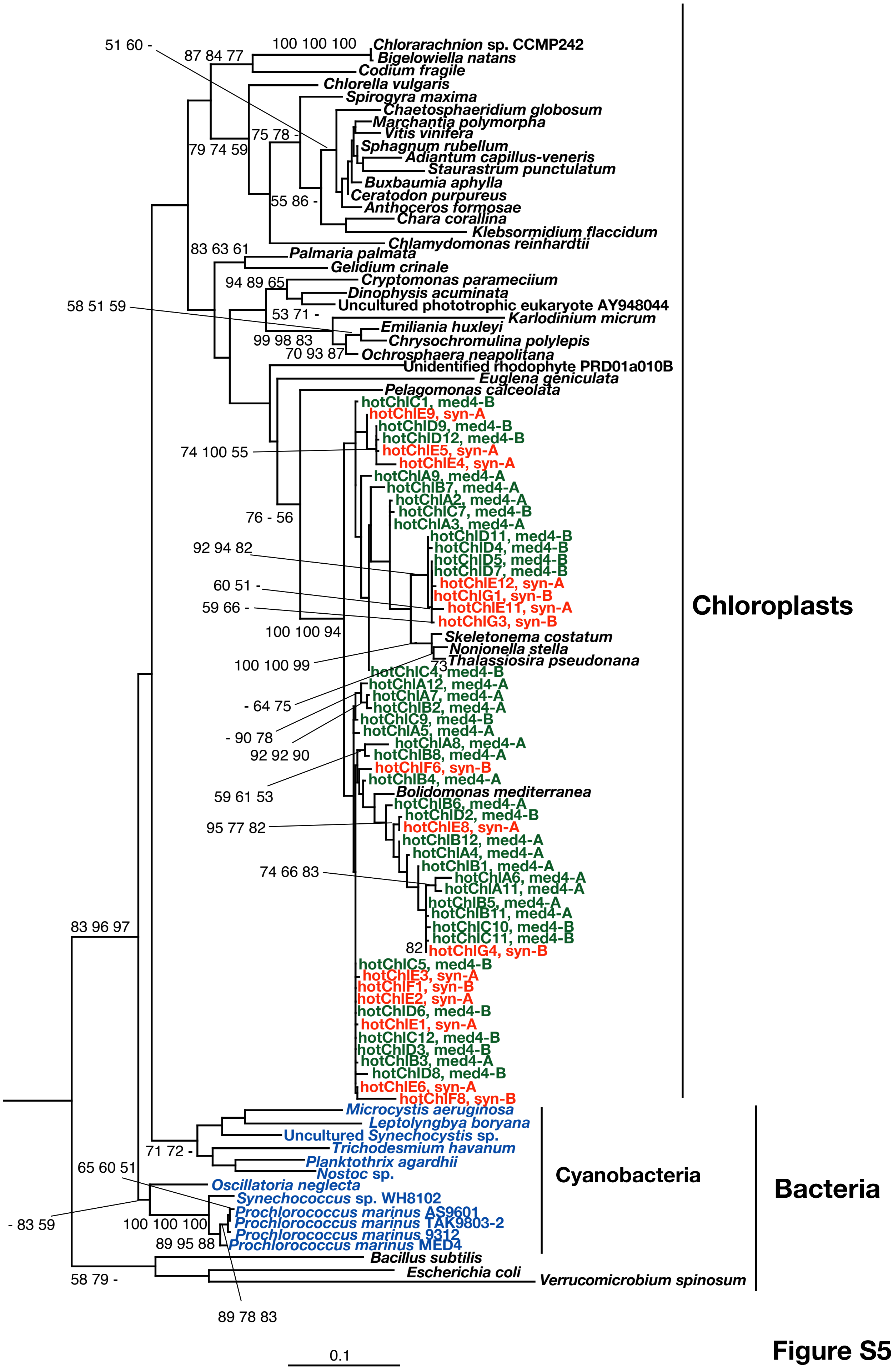

Chlorarachnion sp. CCMP242
Bigelowiella natans
Codium fragile
Chlorella vulgaris
Spirogyra maxima
Chaetosphaeridium globosum
Marchantia polymorpha
Vitis vinifera
Sphagnum rubellum
Adiantum capillus-veneris
Staurastrum punctulatum
Buxbaumia aphylla
Ceratodon purpureus
Anthoceros formosae
Chara corallina
Klebsormidium flaccidum
Chlamydomonas reinhardtii
Palmaria palmata
Gelidium crinale
Cryptomonas parameciium
Dinophysis acuminata
Uncultured phototrophic eukaryote AY948044
Karlodinium micrum
Emiliania huxleyi
Chrysochromulina polylepis
Ochrosphaera neapolitana
Unidentified rhodophyte PRD01a010B
Euglena geniculata
Pelagomonas calceolata
hotChlC1, med4-B
hotChlE9, syn-A
hotChlD9, med4-B
hotChlD12, med4-B
hotChlE5, syn-A
hotChlE4, syn-A
hotChlA9, med4-A
hotChlB7, med4-A
hotChlA2, med4-A
hotChlC7, med4-B
hotChlA3, med4-A
hotChlD11, med4-B
hotChlD4, med4-B
hotChlD5, med4-B
hotChlD7, med4-B
hotChlE12, syn-A
hotChlG1, syn-B
hotChlE11, syn-A
hotChlG3, syn-B
Skeletonema costatum
Nonionella stella
Thalassiosira pseudonana
hotChlC4, med4-B
hotChlA12, med4-A
hotChlA7, med4-A
hotChlB2, med4-A
hotChlC9, med4-B
hotChlA5, med4-A
hotChlA8, med4-A
hotChlB8, med4-A
hotChlF6, syn-B
hotChlB4, med4-A
Bolidomonas mediterranea
hotChlB6, med4-A
hotChlD2, med4-B
hotChlE8, syn-A
hotChlB12, med4-A
hotChlA4, med4-A
hotChlB1, med4-A
hotChlA6, med4-A
hotChlA11, med4-A
hotChlB5, med4-A
hotChlB11, med4-A
hotChlC10, med4-B
hotChlC11, med4-B
hotChlG4, syn-B
hotChlC5, med4-B
hotChlE3, syn-A
hotChlF1, syn-B
hotChlE2, syn-A
hotChlD6, med4-B
hotChlE1, syn-A
hotChlC12, med4-B
hotChlD3, med4-B
hotChlB3, med4-A
hotChlD8, med4-B
hotChlE6, syn-A
hotChlF8, syn-B
Microcystis aeruginosa
Leptolyngbya boryana
Uncultured Synechocystis sp.
Trichodesmium havanum
Planktothrix agardhii
Nostoc sp.
Oscillatoria neglecta
Synechococcus sp. WH8102
Prochlorococcus marinus AS9601
Prochlorococcus marinus TAK9803-2
Prochlorococcus marinus 9312
Prochlorococcus marinus MED4
Bacillus subtilis
Escherichia coli
Verrucomicrobium spinosum
Chloroplasts
Cyanobacteria
Bacteria
100 100 100
87 84 77
51 60 -
79 74 59
75 78 -
55 86 -
83 63 61
94 89 65
58 51 59
53 71 -
99 98 83
70 93 87
74 100 55
76 - 56
92 94 82
60 51 -
59 66 -
100 100 94
100 100 99
73
- 64 75
- 90 78
92 92 90
59 61 53
95 77 82
74 66 83
82
83 96 97
71 72 -
65 60 51
- 83 59
100 100 100
89 95 88
58 79 -
89 78 83
0.1

Figure S5
