# Supplementary material for: Use of stable isotope-labelled cells to identify active grazers of picocyanobacteria in ocean surface waters
Source: Environ Microbiol. 2009 Feb;11(2):512–25. doi: 10.1111/j.1462-2920.2008.01793.x (PMC2702499; doi:10.1111/j.1462-2920.2008.01793.x)
Supplement: Supplementary file 2 [file emi0011-0512-SD2.doc]

**Table S1.** Oligonucleotides used for the amplification of 16S rRNA chloroplast genes from different groups defined based on the ARB tree (SSRef release 90 12.05.2007) for these group of sequences. F, forward primer. R, reverse primer.

Primers’ Group targeted Sequence Expected size

set of amplified

number product in bp

1 Zea and rel.-F GAA GTG GTG TTT CCA GTG GC 351

Zea and rel.-R AAA AGA AGT TCA CGA CCC GT

2 Chlamydomonas and rel.-F ACA CGT CAA CGC ACG AGC TG 821

Chlamydomonas and rel.-R TAG CTA GTT GGT GGG GGT AA

3 Anthocerus and rel.-F TAA GGA GGG GCT TGC GTT TG 218

Anthocerus and rel.-R GTC ATT GCT TCT TCT CTA AG

4 Marchantia and rel.-F CCT TTT CTC AGA GAA GAT GC 813

Marchantia and rel.-R GCG AGG TCG CGA CCC TTT GT

5 Spirogyra and rel.-F TAG TCT CCA CCG CCT GGC CA 485

Spirogyra and rel.-R GGC GGG GGA CCA CCA CTG GA

6 Palmaria_1 and rel.-F CGC CTT AGC TAC GAT ACT GC 89

Palmaria_1 and rel.-R AGA CGA CAG CTA GGG GAG CA

7 Ochromonas and rel.-F CCA CCT GTG TAA GAG GCC GT 581

Ochromonas and rel.-R GGA AGA TCT GAC GTT ACT TG

8 Palmaria_2 and rel.-F CTA CGA TAC TGC ACG GAT CG 136

Palmaria_2 and rel.-R TGG GAA GAA CAC CAG AAG CG

9 Chara and rel.-F GCA CTG AAC GGA TCA AAT CG 712

Chara and rel.-R AAG GAC TTG CCC TTG GGT GG

10 Bryophyta and rel.-F GGA GCG AAA GGA GGA ATC CA 31

Bryophyta and rel.-R ACG CAA GCC CCT CCT TGG GT

11 Chlorella and rel.-F CCC CAG GCG GGA TAC TTC ACG 160

Chlorella and rel.-R GGG AGG AAC ACC AAA GGC GA

12 Chlorarachnion and rel.-F AGC GAG GGG AGA GAA TGG GA 436

Chlorarachnion and rel.-R GCC CAG AAC TTA AGG GGC AT

13 Osmunda and rel.-F AGC AAA AGG GAG GGA TCC GC 398

Osmunda and rel.-R TTG ACA GCG GAC TTA AGG AG

14 Chaetosphaeridium and rel.-F CTT GCG TCT GAT TAT GCT AG 175

Chaetosphaeridium and rel.-R ACC CGT AAG CTT TCT TCC T

15 Skeletonema and rel.-F TTA ACT CAA GTG GCG GAC GG 650

Skeletonema and rel.-R AGT GTT AGT AAT AGC CCA GTA

16 Euglenales and rel.-F GGG GAG TAC GCT TGC AAA AG 153

Euglenales and rel.-R CAT GCA CCA CCT GTG TCT AG
